# Removal of Hazardous Organic Dyes from Liquid Wastes Using Advanced Nanomaterials

**DOI:** 10.3390/ijms25179671

**Published:** 2024-09-06

**Authors:** Francisco Jose Alguacil, Manuel Alonso, Jose Ignacio Robla

**Affiliations:** Centro Nacional de Investigaciones Metalurgicas (CSIC), Avda. Gregorio del Amo 8, 28040 Madrid, Spain; malonso@cenim.csic.es (M.A.); jrobla@cenim.csic.es (J.I.R.)

**Keywords:** nanomaterials, organic dyes, removal, environment

## Abstract

The presence of organic dyes in aqueous environments is extremely hazardous to life due to the toxicity of these compounds. Thus, its removal from these various aquatic media is of the utmost importance, and several technologies are constantly being tested to meet this goal. Among these technologies, various types of degradation and adsorption techniques are typically used, and of the various types of materials used within these technologies, nanomaterials are constantly being developed and investigated, likely due to the various properties that these nanomaterials have. This work reviewed recent developments (in 2023) about the use of these nanomaterials in the treatment of solutions contaminated with these toxic organic dyes.

## 1. Introduction

Organic dyes are chemicals that are commonly used in the textile industry to add color to various products. Therefore, the effluents produced by this industry contain variable concentrations of these harmful dyes, and their removal from these effluents is essential for avoiding environmental issues. Low amounts of dyes, i.e., below 1 ppm, can clearly be seen in some waterbodies, where they can block the penetration of sunlight and inhibit photosynthesis in aquatic plants. Thus, effluents from the textile industry not only change the color of those waterbodies but also affect the habitats of living organisms by decreasing or even preventing sunlight penetration and water re-oxygenation capacity, disrupting aquatic life. In some cases, dyes can contaminate drinking water and thus pose a serious threat to human life due to the carcinogenic nature of some dyes. In addition, continuous contact with these dyes leads to several health problems, such as headache, confusion, vomiting, high blood pressure, shortness of breath, allergic reactions, mental disorders, kidney dysfunction, and damage to the reproductive system, liver, brain, central nervous system, and others [1,2,3,4].

Many types of materials are currently being used to remove these dyes; among them, nanomaterials are gaining considerable attention due to their inherent properties. The importance of the removal of these dyes and the use of nanomaterials for their removal is reflected in several recent reviews about this matter. Table 1 summarizes the different classes of nanomaterials recently reviewed (in 2023). Moreover, the importance of these investigations is highlighted by the publication of additional reviews at the beginning of 2024 (date of this revision). The latest progress in Bi_5_O_7_I-based heterojunctions was reviewed in [5], providing important information about three main areas: synthesis approaches, characterizations, and their applications in the removal of dyes. Another publication reviewed [6] the synthesis methods, physicochemical properties, and different applications of ferric tungstate (FeWO_4_) as smart multifunctional nanomaterials for various water pollutants, including organic dyes; the uses of carbon quantum dots (CQDs) in the degradation of various dyes, as well as the difficulties that still exist and the opportunities that lie ahead, have also been reviewed [7].

Methylene blue dye is commonly used as the standard to investigate some removal procedures, and consequently, a publication reviewed the principles of photodegradation and explored the application of polymer-supported nanomaterials in the removal of this harmful dye [8]. One review paper [9] focused on the usage of carbon nanotubes (CNTs) and conducting polymer (CP: polyaniline and polythiophene)-based nanocomposites for environmental remediation, which also included organic dyes as toxic pollutants. The use of MoS_2_-based nanomaterials for the removal of organic dyes from solutions through adsorption and photocatalytic processes is of interest and has thus been the goal of another recent review [10], as well as the use of nanoparticles to tackle the issue of the photocatalytic degradation and removal of organic dye pollutants from different aquatic environments [11]. It is worth noting that third-generation catalysts, comprising photocatalysts spread on inert solid substrates, such as Ce/TiO_2_ and Al/ZnO, are the latest developments in materials based on nanoparticles.

There are several subclasses of this type of nanomaterials, and they mainly remove organic dyes from the effluents by two types of processes or mechanisms: adsorption and/or (photo)catalysis.

The present work reviewed the most recent literature (papers published in 2023) on the removal of organic dyes from liquid effluents using nanomaterials. This review classified the investigation considering the types of dyes involved in the literature (anionic and cationic) and nanomaterials used for the treatment of both anionic and cationic dyes (a comprehensive list of the reviewed organic dyes is included in the Supplementary Materials).

**Table 1:** Recent reviews (from 2023) on the use of nanomaterials for the removal of organic dyes from solutions.

Nanomaterial	Reference
Heteropolyacids supported on nanocatalysts Nanomaterials Bi-based nanocompounds Graphitic carbon nitride Sr-based nanomaterials Nano-metal oxide-activated carbon TiO_2_-based nanomaterials Nanoparticle-hybridized polyaniline Metal ferrite-based nanomaterials Metal sulfide-based nanostructures Polymeric nanocomposite membranes Metals and metal oxides in pure form Graphene-based nanomaterials Cellulose-based nanocompounds Biomass-synthesized metallic nanoparticles Nanozymes 2D MXene-based adsorbents Ba-TiO_3_ nanomaterials Gum-based nanomaterials Chitosan-based nanomaterials Carbon-based nanomaterials	[12] [13,14,15,16,17,18,19] [20,21,22] [23,24] [25] [26] [27,28,29,30] [31] [32] [33,34] [35,36] [37,38,39,40,41] [42,43] [44,45,46] [47] [48,49,50,51] [52,53,54] [55] [56] [57] [58,59]

## 2. Nanomaterials and Anionic Organic Dyes

### 2.1. Adsorption of Anionic Dyes on Nanomaterials

The adsorption methodology has received much attention due to its cost-efectiveness, high efficiency, and simplicity. For this reason, in recent years, the design and construction of new adsorbents with high adsorption and removal properties is increasing. Porous metal–organic frameworks (MOFs) are one of these adsorbents that have been considered for the removal of hazardous substances due to their remarkable chemical stability in the solvent, their specific surface area, pore channel size, type and number of adsorption sites, electrostatic interaction with adsorbates, and π–π interactions between benzene rings of MOF and aromatic rings of dyes and organic compounds (further comments included in the Supplementary Materials).

Computational investigation was used to study the performance of TiO_2_ in the uptake of Acid Yellow 36 and Acid Orange 6 dyes on (1 1 0) surface of the oxide [60]. The results of these simulations indicated that Acid Yellow 36 was more reactive and had a greater adsorption uptake than Acid Orange 6.

Gelatin-bearing semi-IPN hydrogels containing different concentrations of itaconic acid-based protic ionic liquids (PILs) were prepared to remove Reactive Red 21 and Reactive Blue 19 from water [61]. When the hydrogels contained zero or low PILs concentrations, the adsorption uptake was maximum, being the best-performing hydrogel composition (GelITA1) tested in real wastewater (cotton samples were impregnated with both dyes, and the resulting wastewater was used in the experimentation) with an average 75% removal efficiency of the dyes. Maximum dye uptakes were 106 mg/g and 147 mg/g for Reactive Red 21 and Reactive Blue 19, respectively.

ZnO/C nanocomposites were fabricated at temperatures in the 500–700 °C range. Among all the composites, the ZnO/C-700 sample was the nanomaterial with better adsorption characteristics for the removal of Congo Red from solutions [62]. The maximum upload was near 47 mg/g.

A sol-gel procedure was used to form MgO nanoparticles, which have been used to adsorb Reactive Red 21 [63], with a maximum adsorption loading of 385 mg/g at a pH range of 5–9, and 20 min of contact time. Dye-bearing MgO nanoparticles were regenerated at 500 °C for two hours and re-used with reasonable efficiency (pink color appeared in the solution after the sixth cycle, increasing in color within the seventh cycle). These MgO nanoparticles presented a 98% dye removal from a real textile wastewater (formed as in [61]).

CuFe-LDH/g-C_3_N_4_ (CFL/CN) nanocomposites were prepared and investigated to remove Alizarin Red S, and the results were compared with the derived from the use of CFL/CN nanoadsorbent [64]. Sono-CFL/CN nanoadsorbent was more effective (near 96%) on the removal of the dye in comparison to hydro-CFL/CN nanomaterial (53%). Maximum dye uptake was in the 65–75 mg/g range, which was dependent on the technology used to remove the dye. Sono-CFL/CN lost its initial adsorption capacity under continuous use (Table 2).

A nanostructured composite based on superparamagnetic iron oxide nanoparticles (SPIONs), coated with hydroxyapatite (HAp), has proven to be a good adsorbent against the presence of Congo Red in aqueous solutions [65]. Amongst the various formulations, the composite containing 35 wt% of SPION was selected to adsorb the organic dye. The results concluded that the best efficiency on the removal of the dye were achieved when 0.10 g of SPION/HAp treated 50 mL of a solution containing 50 mg/L of the dye at pH 0 and a contact time of four hours. The maximum Congo Red adsorption uptake using the SPION/HAp adsorbent was determined to be 158.98 mg/g. The composite can be separated from the solution using magnets.

An ion gelation procedure was used to fabricate chitosan nanoparticles, which were used on the removal of anionic dyes (Reactive Yellow 145, Reactive Blue 19, and Reactive Red 195) from solutions [66]. The utilization of polyaluminum chloride (PAC) (80 mg/L) and chitosan nanoparticles (150 mL/L) at pH = 6.6 produced the maximum (92%) color removal efficiency. Thus, with the use of chitosan nanoparticles, the amount of PAC needed to increase the color removal efficiency was reduced. Furthermore, the effect of chitosan nanoparticles on the adsorption of the dyes revealed that the Langmuir type II isotherm equation was followed, with a maximum adsorption capacity of 1100 mg/g.

Reference [67] investigated the production of amorphous biochar from breadfruit leaves, a product that has been used to remove Congo Red from solutions. The average size of the char was 190 nm; however, after the adsorption of the dye, the average size increased to 460 nm. The adsorption of Congo Red was studied under different dye concentrations (5–50 mg/L), reaction times (30 min–four hours), pH (6–9), and char dosages (0.5–2 g/100 mL). RSM-BBD design results showed maximum removal efficiency (99.96%) at pH 6.37, dye concentration of 45 mg/L, time 105 min, and biochar dosage of 1.92 g. Adsorption data fitted well to the Langmuir Type-II isotherm, with a maximum upload of almost 18 mg/g.

Another nanobiomaterial was used in the next study. A nanobiosorbent was fabricated via microwave crosslinking of nanosilica gel with banana peels nanobiochar (BPNB) and chitosan hydrogel (Chit Hgel) to produce BPNB-NSiO_2_-Chit Hgel nanobiosorbent (22.48–26.23 nm) [68]. The influence of nanobiosorbent dosage (1–50 mg), pH (2–12), reaction time (1–45 min), dye concentration (5–100 mg/L), temperature (15–60 °C), and interfering salts on the adsorption of Congo Red was investigated. Maximum dye uptake was about 58 mg/g. Removal of the dye (92.8–95.0%) from tap, sea, and wastewater can be accomplished.

Zinc ferrite (ZnFe_2_O_4_, BET surface area 85.88 m^2^/g) and copper ferrite (CuFe_2_O_4_, BET surface area 41.81 m^2^/g) nanoparticles were formed, and used as adsorbents, in the removal of Alizarin Yellow R, Thiazole Yellow G, Congo Red, and Methyl Orange from industrial wastewater [69]. The use of acidic pH values favored the removal of the dyes, the maximum monolayer adsorption capacities being higher when using zinc ferrite than when using copper ferrite (Table 3).

In this work [70], multifunctional Fe_3_O_4_@N-C (nitrogen-doped carbon) nanocomposites with hollow porous core–shell structure were formed by different procedures. These included i) a hydrothermal method, ii) polymer coating, iii) thermal annealing process in nitrogen and iv) etching step in HCl medium. Fe_3_O_4_@N-C nanocomposites etched by HCl for different lengths of time, especially Fe_3_O_4_@N-C-3 (30 min of etching time) nanocomposites, adsorbed Methyl Orange (98.7% after 100 min).

Sodium alginate/gellan gum and polyethyleneimine were utilized to fabricate a material with 3D hierarchical porous architecture and used in the removal of Congo Red from waters [71]. The aerogel showed an adsorption uptake of 3 g/g, selectively separate Congo Red in the presence of other dyes from binary systems: Congo Red plus Amaranth (anionic dye), Safranine T (cationic dye), Methyl Orange (anionic dye) or Rhodamine B (cationic dye). From single-dye solutions, the affinity of the aerogel from the dyes followed the given order: Congo Red > Rhodamine B > Methyl Orange > Amaranth > Safranine T. After three cycles, the adsorption capacity decreased by 14.8%, maintaining an adsorption capacity of approximately 0.5 g/g (0.65 g/g in the first cycle).

The same dye as above, Congo Red, was removed from the solution using green pea peel biochar (GPBC) and zinc oxide green pea peel biochar nanocomposite (ZnO/GPBC, particle size 14.58 nm) [72]. The experimental conditions used in the investigation were as follows: dye concentration (50–250 mg/L), GPBC and ZnO/GPBC dosage (50–250 mg/100 mL), pH (2−12), temperature (20–60 °C) and time (0–90 min). The results showed that ZnO/GPBC was more effective at removing the dye than GPPBC (98% versus 90%, respectively) under the optimized conditions. The monolayer adsorption capacities were 114.94 mg/g and 62.11 mg/g for ZnO/GPBC and GPBC, respectively. In both cases, there was a decrease in the dye removal capacity with the number of cycles: ZnO/GPBC (98.4% in the first cycle against 88.8% in the fifth cycle), GPBC (90.4% first cycle versus 76.2% fifth cycle).

The next two references also used Congo Red as target to investigate the benefits of using their respective nanomaterials. The removal of Congo Red from mimic wastewater was used to evaluate the properties of different composition ratios of CMC-nZVI (nano zero-valent iron coated with carboxymethyl cellulose) [73]. At an initial dye concentration of 500 mg/L, the increase in CMC contents favored the decolorization process. The removal efficiency was near 100% at the CMC:Fe^2+^ ratio of 0.004 within 10 s after the addition of CMC-nZVI to the dye solution, whereas the optimal decolorization rate (90%, with an uptake capacity of near 8 g/g) was observed when the iron concentration was 1 g/L. The above results were attributable to the break of the chromophore group (-N[dbnd]N-), responsible for the color of the dye, to N–H bonds.

F-Ln (Ln = Ce, Pr and Nd) nanosheets, provided by surface acetate groups, were prepared through a hydrothermal synthetic methodology at room temperature and pressure, and used in the adsorption of Congo Red and Coomassie Brilliant Blue G-250 anionic dyes [74]. The maximum Langmuir adsorption capacities for the respective two dyes were 4334 mg/g and 2827 mg/g. Both dyes were loaded onto the adsorbent via its exchange with the surface acetates groups of the F–Ce nanosheets. Furthermore, these nanosheets presented a salt tolerance in saturated NaCl medium or elevated concentrations of NaO_4_, K_2_CO_3_ and NaNO_3_ in the solutions.

### 2.2. (Photo)-Catalytic Degradation of Anionic Dyes Using Nanomaterials

Another useful methodology to remove organic dyes from effluents is (photo)-catalytic degradation; that is, at the same time in which the dye is removed from the effluent, it is destroyed or degraded by this methodology. Commonly used methods of synthesizing photocatalytic nanomaterials are solution-based, thermal-based, vapor-phase, transport processes and microwave-assisted methods. Complexing agents, polymerizing agents and surfactants enable more intricate control of these properties through slowing seed nuclei formation. Nanoparticles have been effectively used in catalytic applications, and the size of the particles is one of the vital factors versus bulk counterpart. CuO nanoparticles has low band gap energy, which makes it preferable material in photocatalytic reactions (further comments included in the Supplementary Materials).

By means of *Moringa stenopetala* seed extract, copper oxide nanoparticles (average particle size 6.6 nm) were formed and used to degrade Congo Red and Alizarin Red S [75]. After two hours of exposure to irradiation and varying experimental conditions, Congo Red degraded by nearly 98%, whereas the degradation of Alizarin Red S reached 95%. The continuous use of the catalyst for five cycles was also investigated, and in both cases, the adsorbent lost its effectiveness after each cycle.

Under a chemical precipitation procedure, zinc oxide (ZnO), copper ferrite (CuFe_2_O_4_) and a zinc oxide–copper ferrite binary composite were prepared, followed by the formation of a ternary composite (ZnO-CuFe_2_O_4_-CNTs) (CNTS: carbon nanotubes) [76]. These compounds were used to search the photocatalytic degradation of Congo Red under sunlight exposure, reaching 82% dye degradation in two hours. The ZnO-CuFe_2_O_4_-CNTs nanocomposite had better degradation efficiency than the pure oxides and their binary composite.

Another commonly investigated dye, as being Methyl Orange, was used in the next three references. Bi_2_O_3_-NiO heterojunction was formed from the respective Ni(II) and Bi(III) nitrates and used to degrade Methyl Orange under visible-light exposure [77]. The photocatalytic degradation of the dye reached rates of 46% (nickel oxide), 67% (bismuth oxide) and 100% (mixed nickel-bismuth oxide). Transfer of positive holes and electrons and the separation being the responsible for the better activity of the mixed oxides heterojunction, which apparently can be recycled several times, as written in the published Abstract, However, these data did not appear in the publication.

Bi_2_WO_6_ nanomaterials, containing a chelating agent (EDTA), were used to degrade Methyl Orange and Congo Red in the presence of NaBH_4_, being the catalytic efficiency of Bi_2_WO_6_ surpassed by that of the EDTA-Bi_2_WO_6_. This catalyst could be recycled up to five times without undergoing a significant loss in activity [78].

Carbohydrate-conjugated Ag, Au and Ag-Au alloy biocompatible nanoparticles (14.5, 11.7 and 16.3 nm respective average particle size) had been fabricated through bio-reduction of the metal salts in the presence of *Lobaria retigera* (high altitude lichen) [79]. These nanoparticles acted as catalysts for the degradation of Methyl Orange.

In order to control its characteristics, β-cyclodextrin and honey were used as surfactants during the synthesis of ZnO nanoparticles, which were coupled with silver to form ZnO-xAg, ZnO-C-xAg and ZnO-H-xAg binary structures with typical crystalline sizes of 9.38 nm [80]. The photodegradation of Bromophenol Blue showed increased photoactivity upon modification with cyclodextrin, honey, and silver nanoparticles. Dye photodegradation was near 100% by the use of ZnO-H-3Ag (3 wt% silver).

Ti_3_C_2_ MXene and its nitrogen-doped derivative (N-Ti_3_C_2_ MXene) were used in the removal of Methyl Orange from waters [81]. The results indicated that the catalytic efficiency of N-Ti_3_C_2_ MXene increased nearly ten times in comparison with the corresponding of the Ti_3_C_2_ MXene nanomaterial. N-Ti_3_C_2_ 1:8 MXene composition performed best, since the degradation of a 20 mg/L dye solution reached more than 98% after twenty minutes of UV lamp irradiation. The effectiveness of this material was attributable to the presence of more abundant surface functional groups, improving the charge transfer efficiency and enhancing the electrical conductivity of the catalyst.

Ag/Mn–ZnO nanomaterial (4–5 nm) was used to remove Methyl Orange and Alizarin Red S from solutions; both dyes degraded by nearly 70% after 100 min of reaction time [82]. However, and in the case of Alizarin Red S, the nanomaterial did not maintain its degradation efficiency under continuous use (five cycles). 

Two-dimensional borate anions B_4_O_5_(OH)_4_^2−^ intercalated layered double hydroxide (ZnGa-BLDH) nanosheets (5–6 nm) were investigated in the photodegradation removal of Congo Red from an aqueous environment [83].

The aqueous extract of *Ulva lactuca* and ZnO nanopowders were used to fabricate ZnO@C nanocomposites, material that was investigated to degrade Congo Red under UV and visible light exposure [84]. This nanocomposite degraded the dye better than the pristine zinc oxide nanopowder.

Undoped TiO_2_ and Fe-doped TiO_2_ materials, prepared via sol-gel and precipitation procedure, were used as photocatalysts to remove Methyl Orange under UV-A and visible-light irradiations [85]. The photocatalytic experiments revealed an increase in the photocatalytic activity of 0.5 wt% Fe-doped TiO_2_ composition, and only under UV-A light, compared to the rest of the prepared nanomaterials. This increase was due to the efficient charge separation of electrons and holes.

Another nanobiomaterial was used in the next reference to degrade the well-known Methyl Orange dye. Iron oxide nanoparticles (IONPs), composed of amorphous FeOOH and FeII/III-polyphenol complexes with extracts of the plant *Camellia sinensis*, were used to remove Methyl Orange from solutions [86]. The nanoparticles, as heterogeneous-Fenton catalysts, were used for the removal of the dye. The best results were obtained at pH values around 3.

Graphene oxide nanosheets-yttrium oxide (Y_2_O_3_)–silicon oxide (SiO_2_) ternary nanostructures were fabricated to investigate its photo-degradation activity against the presence (20 ppm) of Methyl Orange in aqueous solutions [87]. The photo-degradation of the dye improved from 1.17% for Y_2_O_3_/SiO_2_ nanostructures to 52% when the concentration of the graphene oxide nanosheets reached 15 wt% and 45 min of reaction time.

Pristine Fe_2_O_3_, 1 wt% metal (Ag, Co, and Cu)-doped Fe_2_O_3_ nanoparticles (NPs) were fabricated using a hydrothermal and wet impregnation method [88]. The nanoparticles were employed as photosensitizers for the degradation of Reactive Red and Orange II dyes under sunlight irradiation. The synthesized 1 wt% Ag–Fe_2_O_3_ (AgF) NPs had the best catalytic properties, compared with pristine Fe_2_O_3_ and CoF and CuF, in degrading (98.32%) both dyes within 120 min of reaction time.

The photocatalytic properties of polymeric g-C_3_N_4_ layers coated with various dosages of erbium oxide nanoparticles were investigated through the oxidation of Orange G under visible-light exposure. The nanoparticles maintained their activity above 95% for up to three cycles of reusability [89].

ZnO-ZnS-CdO-CdS quaternary core–shell nanocomposites (NCs) were fabricated using *Ricinus communis* as a stabilizing agent and hydrazine hydrate as a reducing agent [90]. The nanocatalysts were used in the degradation of Congo Red and Methyl Red dyes, being active both in the degradation of single or a mixture of the dyes.

The removal of Congo Red, under UV light in the presence of H_2_O_2_, and the photocatalytic activity of copper oxide nanoparticles (CuONPs, 25–90 nm size) were investigated [91]. The UV light decomposed H_2_O_2_ with the formation of ·OH radicals, which improved the degradation of the dye. This degradation occurred by reaction of the dye with the radicals, though other reactions of the dye with, i.e., a positive hole (h^+^_vb_), contribute to the elimination of this harmful compound.

A one-step laser technology was used to fabricate doped boron and boron and nitrogen co-doped, which highly reduced graphene oxide and multiwalled carbon nanotubes/Ni oxide and Ni hydroxide nanohybrid layers [92]. These materials were employed to investigate their properties regarding the photocatalytic removal of Methyl Orange. The best photocatalytic activities resulted for boron and nitrogen co-doped materials formed by the use of H_3_BO_3_ and urea as boron and nitrogen precursors, respectively; however, the degradation efficiency failed after continuous use: 80% in the first cycle and 3% in the third cycle.

A direct thermal polymerization procedure was utilized in the fabrication of 1D/2D g-C_3_N_5_/g-C_3_N_4_ heterojunction (1D/2D-N_5_/N_4_) used in the removal of Methyl Orange from wastewater [93]. One-dimensional/two-dimensional-N_5_/N_4_ had one-dimensional g-C_3_N_5_ nanorods and two-dimensional g-C_3_N_4_ nanosheets. The photocatalytic degradation of the dye reached a rate of 99.6%, maintaining 92.20% after five cycles.

Attapulgite (ATP) mineral nanomaterials grafted with magnetic Fe_3_O_4_ nanoparticles, with a further modification with polyethyleneimine (PEI), served in the fabrication of functionalized magnetic attapulgite composites (ATP-Fe_3_O_4_-PEI), which was utilized for the removal of Congo Red from wastewater [94]. The removal of the dye by means of the nanocomposite was attributed to a catalytic degradation process. However, without photoirradiation and the presence of oxidants in the solution, the system formed H_2_O_2_ and hydroxyl radicals (·OH) to catalytically degraded dye molecules. Moreover, the removal of the dye was further increased by adsorption on the composite material through electrostatic attraction, hydrogen bonds, and π–π interaction. Under two removal tests, the removal rate of the dye exceeded 95%, corresponding to a removal capacity of 943.77 mg/g, at pH 3 and 25 °C. This composite presented removal effectiveness and uptake capacity exceeding 82% and 799 mg/g, respectively, after four cycles.

The efficiency of Mn-20wt% Ni and Mn-30wt% Ni particle powders in relation to the degradation of Reactive Black 5 as function of different experimental variables was investigated [95]. Mn-20wt% Ni particles presented better degradation efficiency and faster rate of reaction than Mn-30wt% Ni material.

The metal-assisted chemical etching (MACE) technique was used to produce SiNWs-NiNPs/NiONPs [96]; these nanoparticles were used in the photocatalytic assisted degradation of Methyl Orange from an aqueous solution. The degradation of 20 ppm of the dye had an efficiency of 66.5% after three hours and under UV exposure.

A manganese oxide (MnO_2_)/silicon carbide (SiC) nanostructure-doped polystyrene (PS)–polycarbonate (PC) blend was used to investigate its ability to degrade Methyl Orange [97]. The results indicated that the use of PS–PC/MnO_2_–SiC nanocomposites under UV exposure removed the dye color with an efficiency of approximately 54% within 90 min, and using the 5.2 wt% MnO_2_/SiC NPs formulation.

Carbon dots with diameters of 4.9 ± 1.5 nm and 4.1 ± 1.2 nm were fabricated from nitric and sulfuric acids, respectively, and *Ilex paraguariensis* as raw material [98]. These dots were used to fabricate magnetite-containing nanocomposites, which were nanocomposites utilized to investigate the photocatalytic degradation of Methyl Orange. At pH 6.2, 8.5 ppm dye concentration and 100 ppm nanomaterials doses, all the above nanocompounds presented catalytic activity, allowing the removal of up to 98% dye under visible irradiation (400 nm) and seven hours of reaction.

Ionic liquids (ILs) were also used to formulate nanostructures to degrade dyes. Cetyltrimethylammonium bromide (IL) surfactant-assisted hierarchical TiO_2_ heterojunction was doped with europium to form a nanostructure with crystallite sizes in the 4–10 nm range [99]. Further, Congo Red dye was photocatalytic degraded within the above materials in the presence of UV light, increasing the degradation rate with the reaction time. After 75 min, the efficiency of the dye degradation reached 97%; it was also concluded that the degradation is dependent on the dispersion of the nanomaterials.

Multifunctional surface-modified CuO nanomaterials were used for the degradation of AR88 dye. At pH 11, the nanomaterial degraded by approximately 95% of the dye under UV exposure, and utilizing a 1 g/L nanomaterial dose [100].

Zinc oxide/copper oxide nanocomposites (ZnO/CuO NCs) presenting various proportions of binary oxides were investigated for their degradation of Congo Red [101]. Experiments carried out on dye-bearing water and real textile wastewater under visible light irradiation by a 0.5 M ZnO:0.5 M CuO nanocomposite showed both acting as catalysts by tuning the doping concentration of copper oxide in ZnO.

Polymethyl methacrylate (PMMA)-polycarbonate (PC)/antimony oxide (Sb_2_O_3_)-graphene oxide (GO) nanocomposites films were fabricated [102]. These nanomaterials were used in the photodegradation of a 20 ppm Methyl Orange solution. This degradation was due to a rise in electron–hole pairs and a reduction in fix replication.

This reference investigated a ceramic-based nanomaterial catalyst (a reduced graphene oxide-ZnS, rGO-ZnS) for the oxidative photocatalytic degradation of Naphtol Blue Black dye solution under exposure to sunlight [103]. The dye was degraded (93.7%) under exposure to direct solar light.

A SiO_2_/g-C_3_N_4_ photocatalyst was fabricated, fixing the amount of SiO_2_ and varying the concentration of graphitic carbon nitride in the ratio (1:x, where x = 1, 2, 3); these nanocomposites were used to investigate their photocatalytic properties to degrade Auramine O and Xylenol Orange dyes [104]. The results showed that SCN_2_ (x = 2) nanocomposite improved the photocatalytic activity under visible light exposure with degradation efficiencies of 70% and 84.6% for the respective above dyes; these results are attributable to the large surface area and the electron–hole separation rate that the nanocomposites presented.

Bismuth oxychloride (BiOCl) nanoplatelets and lignin-based biochar were used as precursors to fabricate composites formed by bismuth oxyhalide nanoparticles, and the simultaneous photocatalytic and adsorptive efficiency of the Biochar–BiOCl (%BC-BiOCl) composites on the removal of Methyl Orange was investigated [105]. Maximum dye removal and degradation (100%) was reached under the following experimental conditions: composite dose of 1.39 g/L, initial dye concentration of 41.8 mg/L, pH of 3.15, and one hour of reaction under light exposure. Similarly to other nanomaterials, there was a loss in efficiency after various cycles; in this case, the efficiency dropped from 99% in the first cycle to 63% in the fifth cycle (1 g/L of 15BCPC dosage, 30 mg/L initial dye concentration at pH 5).

1-hexyl-3-methyl-2-(2-oxo-ethyl)-3H-imidazol-1-ium bromide (HMIB) ionic liquid embedded on CdO-TiO_2_ (HMIB-CT) hybrid nanomaterial was used in the removal of Tripan Blue dye from solutions [106]. Complete dye removal under natural sun beam irradiation, required for photocatalytic activity, was accomplished at pH 7 and one hour of reaction time.

After the formation of the magnetic photocatalyst *Scendesmus* sp./Fe_3_O_4_/TiO_2_, its sonophotocatalytic properties with respect to the degradation of the Red195 dye were investigated [107]. At a pH of 5, photocatalyst dosage of 100 mg, initial dye concentration of 100 mg/L, ultrasound power of 38 W, and an exposure time of 20 min, the maximum Red195 removal efficiency (100%) resulted. After five cycles of recycling, the degradation efficiency remained above 95%. Experiments on scavenging indicated that electrons (h+) and OH^−^ groups were necessary to act as decomposition reagents.

### 2.3. Remarks to the Use of Nanomaterials in the Removal of Anionic Dyes from Solutions

While Section 2.1 and Section 2.2 showed that two approaches are being used by scientists to remove anionic dyes from solutions—adsorption or (photo)-catalysis—Table 4 summarizes the various anionic dyes object of these investigations. From the number of references, it is clear that the (photo)-catalytic degradation of organic dyes is the methodology of preference compared to the use of adsorption–elution in achieving this environmental task. In any case, it is difficult to compare results due to the various experimental conditions used in each reference. Using (photo)-catalysis, the degradation of the dye occurs within a few minutes or hours (see Table 5), whereas in the case of adsorption procedures, dye uptakes vary from a few mg/g to the g/g order (see text and also next Table 10).

From the above, just one reference, [69], utilized real industrial wastewater in the investigation; also, this table shows that Congo Red and Methyl Orange are the most investigated anionic dyes.

## 3. Nanomaterials and Cationic Dyes

### 3.1. Adsorption of Cationic Dyes on Nanomaterials

Obviously, adsorption is a useful methodology in removing cationic dyes from effluents, though some of the characteristics of the adsorbent used here must be different from the adsorbents used in Section 2.1, due to the different characteristics (charge sign of the active group) of cationic dyes with respect to anionic dyes. Thus, it is somewhat expected that in these cases, cationic dyes will be removed from the effluent at pH values higher than the value of the isoelectric point presented by the given adsorbent.

Two different processes (Calix-MNPs and Calix-Si-MNPs) were used to modify magnetite nanoparticles with p-sulfonatocalix [13]arene and were used for the removal of Crystal Violet from aqueous solution [108]. Calix-Si-MNPs showed a better adsorption capacity than Calix-MNPs, attributable to the modification on the surface of magnetite nanoparticles through a spacer. The nanoadsorbent can be recovered using a magnet; however, there was a considerable loss of capacity for up to four cycles of adsorption (pH 7)-desorption (water plus acetone).

In this work [109], amorphous and crystalline products based on Zr, Mg, and Mn were fabricated through the Pechini sol–gel procedure and used to remove Basic Fuchsin dye from solutions. Samples were fabricated before calcination was amorphous, whereas the samples calcined at 500 °C or 700 °C presented mean diameters of 45.16 nm and 76.28 nm, respectively. Table 6 shows that increasing the temperature of calcination the dye uptake onto the nanomaterials decreased. Maximum dye uptake varied in the 93–240 mg/g concentration range.

Succinic anhydride modifying the surface of native cellulose nanocrystals was used to adsorb Golden Yellow and Methylene Blue. No data about the desorption step were given in the work [110].

Bionanocomposites formed of chitosan grafted by various monomers, such as acrylamide, acrylic acid, 4-styrene sulfonic acid, and hybrid nanoparticles of graphene oxide/titanium dioxide nanoparticles (GO@TiO_2_-NPs), were fabricated and used in the adsorption of Basic-Red 46 [111]. The removal of the dye from the aqueous solution followed the pseudo second-order kinetics and Langmuir isotherm models, with maximum upload in the 43–79 mg/g range.

Food industry waste hazelnut shells were used as precursors to prepare, via impregnation with ZnCl_2_ followed by chemical activation with KOH, porous carbon materials to be used as adsorbents of a Methylene Blue wastewater [112]. The Fusso effect, which can reduce the size of the dye molecules, increased the adsorption properties of the carbon. The material showed a dye uptake of 694.03 mg/g, which can be increased to 882.46 mg/g in 0.1 M NaCl medium.

Calcium silicate nanopowders were fabricated from marble sawing dust and silica fume using a microwave irradiation-assisted route [113]. These nanomaterials were investigated in the removal of Malachite Green from solutions in the pH 2–12 range, with best results yielded (near 100% removal) at a pH of 12. Adsorption data fitted well with the linear form of the Freundlich isotherm. Dye desorption was investigated by the use of acetone; after continuous adsorption–desorption cycles, it was shown that after six cycles, the adsorption efficiency decreased from 100% (first cycle) to 78% (sixth cycle). This decrease is attributable to the decrease in the number of the surface-active sites after continuous use.

Under the polyol process, FePt nanoparticles with a size of less than 2 nm were uniformly distributed over the surface of hexagonal boron nitride nanosheets. These materials were used in the adsorption of Methylene Blue, Methylene Violet and Brilliant Green [114]. The removal of the dyes from the solution depended on the composition of the nanomaterial used in the experimentation and the annealing temperature (Table 7). However, no data about the desorption step were provided in the published manuscript.

A nanomanufacturing process, which included the treatment of α-chitin nanocrystals (ChNCs) by electron-beam irradiation, EBI-induced ChNCs, with nano-sized and rod-like morphology with tunable lengths averaging 259–608 nm and uniform widths of 12–16 nm, was developed [115]. The anionic groups presented in the nanomaterials reacted with Toluidine Blue O dye via electrostatic attraction, forming hydrogels, which were self-supporting after centrifugation. No desorption data were included in the manuscript.

Nano-sized polylactic acid was decorated on the surface of graphene oxide to investigate it performance in the removal of Methylene Blue from an aqueous solution [116]. Optimum adsorption parameters were as follows: adsorbent dosage of 0.5 g/L, pH 4, reaction time of two hours, and 45°C. Data were fitted with the Langmuir isotherm with maximum uptakes in the 104–224 mg/g range. Experimental results showed that the loading capacity of the polylactic acid-graphene oxide nanoadsorbent increased by approximately 70% in comparison with that of sulfur oxide. The nanoadsorbent presented a loss of near 27% adsorption efficiency after five cycles.

Using the co-precipitation method, superparamagnetic iron oxide nanoparticles (SPIONs) with sizes of 13.6 ± 1.9 nm were synthesized [117]. Further, the surface of SPIONs was modified by polyvinyl alcohol, chitosan, and graphene oxide, and the modified nanomaterials were used to adsorb Methylene Blue. Removal efficiency at pH 7 reached near 87% after 13 days, and the highest dye-loading capacity was 3.6 mg/g, while the highest loading amount achieved was 36.4 mg/g. The cumulative desorption capacity of the adsorbent at pH 3.8 was at its maximum at almost 90% after 30 days.

The next two references investigated the removal of Methylene Blue dye. Tin oxide nanorods and SnO_2_/MoO_3_ nanocomposite were fabricated via the use of the leaf extract of *Magnifera indica*, the presence of polyphenols in the extract facilitated the reduction in the precursor metal salt to the corresponding oxide [118]. The catalytic properties of the above materials were investigated on the degradation of Methylene Blue. The photocatalytic efficiency of the mixture of oxides improved to 73% as compared to that of SnO_2_ (69.0%).

In [119], Zn-modified biochar obtained from zinc carbonate and Jerusalem artichokes straw was used to explore its effect on the adsorption of Methylene Blue. Compared with the original biochar, the specific surface area (1037.17 m^2^/g) of the Zn-modified biochar by pyrolysis at 800 °C (Zn-BC 800) increased by approximately 22 times. The adsorption of Methylene Blue with Zn-BC 800 followed the pseudo-second-order kinetic model. At 45 °C, Zn-BC 800 had a maximum adsorption loading of 477.13 mg/g. Hydrogen bond and π–π interaction were the main factors responsible for the dye adsorption onto Zn-BC 800. The adsorbent lost its initial dye uptake of near 420 mg/g until 350 mg/g in the fifth cycle; in these series of experiments, anhydrous ethanol and distilled water were used as eluent.

Yellow phosphorus slag was used to develop a SiO_2_ matrix material, which was loaded with MgO, and then the formed composite was investigated as adsorbent of Malachite Green dye [120]. These investigations demonstrated that when using this composite, a maximum dye adsorption rate of 97.72% was reached, with a dye uptake of 115.64 mg/g; experimental data responded well to the Freundlich isotherm. In this case, 0.1 M sodium chloride medium was utilized to desorb the loaded dye, and after four cycles of continuous use, there was a slight decrease in the adsorption efficiency.

### 3.2. Nanomaterials—Forming Membranes for the Removal of Cationic Dyes

Membrane-separation technology is also employed for the treatment of effluents generated by textile dyeing processes. During the filtering process, the micropores included in the membrane filter effectively separate the organic compounds from the effluent by utilizing selective membrane permeability, which can be increased by the use of selective chemicals towards prefereed dye(s).

A ZIF-67/SA@PVDF (ZSA3@PVDF) mixed matrix membrane had been produced by incorporating silicon aerogel (SA) and zeolitic imidazolate framework material 67 (ZIF-67) nanoparticles in a polyvinylidene fluoride (PVDF) membrane [121]. By the use of this membrane, the Methylene Blue removal rate exceeded 99% when filtrating 100 mL of a 5 mg/L dye solution. The continuous use of the membrane showed that its removal efficiency was maintained above 98% after three cycles of the dynamic adsorption process.

### 3.3. (Photo)-Catalytic Degradation of Cationic Dyes Using Nanomaterials

Pure Fe_2_O_3_ (FO with 46 nm particle size) and a series of 5%, 10% and 15% Ni-doped Fe_2_O_3_ (5-NFO, 10-NFO and 15-NFO) nanoparticles (19 nm particle size) were synthesized to investigate the effect of nickel concentration on the photocatalytic activity on Methylene Blue degradation [122]. The highest dye degradation had been obtained using 10-NFO nanoparticles and 100 min of reaction time. The optimal concentration of 10-NFO accelerated the photoreduction of the dye due to the photocatalyst characteristics to trap electrons.

Pristine Fe_2_O_3_ hematite and Nb-doped Fe_2_O_3_ nanostructures with different doping concentrations of 5%, 10%, and 15% of niobium were used to investigate the degradation of Methylene Blue [123]. After 100 min of exposure to visible light, the highest level of dye degradation was attained by 10%-NFO composition. The photocatalytic properties of doped Fe_2_O_3_ were attributed to a decrease in band gap energy.

Mn_4_(P_2_O_7_)_3_ nanoflakes were synthesized and used to remove Methylene Blue from water [124]. The electrostatic interaction between the negatively charged group of the nanoflakes and dye cationic molecules determined the adsorption process; further, the dye was degraded via a heterogeneous catalytic process performed at basic pH in the presence of H_2_O_2_ and reactive oxygen species production. After 30 min and using an initial dye concentration of 40 mg/L, the degradation rate reached 99.2%.

Rhodamine B was degraded by a photocatalytic process under direct sunlight by the use of sol-immobilization synthesized monometallic (Pd and Au) and bimetallic (Pd-Au) hybrid nanomaterials supported on reduced graphene oxide (rGO) [125]. Bimetallic Au-Pd-rGO composites had better catalytic efficiencies than mono-metals-rGO nanomaterials. Under the best formulation (Au0.75-Pd0.25/rGO), the degradation efficiency reached 98%, whereas the nanocomposite can be used for five cycles, maintaining 89% of its activity.

The degradation of Methylene Blue and Rhodamine B has been investigated using cadmium sulfide nanoparticles (CdSNPs, average size less than 20 nm) encapsulated by natural extract [126]. These nanoparticles were synthesized via green chemical reduction route that uses natural stabilizers such as rice water, papaya fruit extracts and precursors such as cadmium chloride, cadmium nitrate, and cadmium sulfur.

Cerium-doped cobalt-magnesium ferrites Co_0.7_Mg_0.3_Ce_x_Fe_2x_O_4_ were prepared and used to investigate their potential as degrading agents against Methylene Blue [127]. The nanocomposite with x = 0.1 presented a degradation efficiency of near 95.5% in one hour, with free radicals O_2_^·−^and OH^.^ acting as the active species to promote dye degradation, which produced CO_2_ and water as final products. This material presented a near constant recyclability efficiency after five cycles (95.5–94.8%).

The next investigation used ZnO–NPs from the milky sap of *Calotropis procera* parts to degrade Malachite Green and Methylene Blue under UV-light exposure [128]. The degradation efficiency reached 85.3% (Malachite Green) and 86.3% (Methylene Blue), in both cases increasing the efficiency from pH 4 to pH 10.

A soft nanocomposite hydrogel, comprising a pyrene-based chiral amphipath with an amino acid (l-phenylalanine) core with pendant oligo-oxyethylene hydrophilic chains and sulfur oxide, was used in the degradation of Methylene Blue and Rhodamine B [129]. Experimental results indicated that the removal efficiency was better in the case of Methylene Blue than Rhodamine B.

Montmorillonite K30 nanosheets were decorated with *Carrisa edulis* fruit extract capped spherical shape Co_3_O_4_ nanoparticles (size of 11.25 nm) to degrade methylthioninium chloride (Methylene Blue) [130]. Attributable to the generation of OH and O_2_ radicals, the 20% loaded Co_3_O_4_ on MK30 nanocomposites had the best photocatalytic performance (near 98%) on exposure to visible light.

0.2CoFe_2_O_4_/0.8TiO_2_-5%La nanocomposite was prepared by a co-precipitation and hydrothermal process, to be used as a high-activity photocatalyst for the degradation of Methylene Blue under visible light irradiation [131]. This dye was degraded by 99% after 50 min of exposure time to the light.

Pure phased rare earth-doped ZnO nanoparticles were fabricated for the degradation of Malachite Green and Crystal Violet [132]. For these dyes, the maximum degradation efficiency was near 98% when dysprosium doped ZnO nanoparticles were used to degrade the dyes. A tailored band gap and the presence of defects, which helped in the creation of a reactive species, was the main factor responsible for the dyes’ degradation.

Ionic mesoporous organosilica (IMOS) forming a polyethylene glycol (PEG)-linked bis-imidazolium chloride framework was prepared for the selective recovery of tungstate ions from wastewater and its further use as a photocatalyst against the presence of Rhodamine B in waters [133]. Under column experiments, a maximum recovery of tungstate ions (123 mg/g) was reached using a bed height of 3 cm and a flow rate of 3 mL/min. The use of this W(VI)-immobilized IMOS (W-IMOS), oxidized Rhodamine B through successive adsorption (30%) in the dark and caused photocatalytic degradation (66%) under UV-vis light irradiation.

Nanomaterials containing the SrMoO_4_/g-C_3_N_4_ heterostructure were synthesized in a single step by the sonochemical method at controlled temperatures; further, these nanomaterials were used to degrade some cationic dyes [134]. The results of the photocatalytic investigations showed that the insertion of CN promoted photocatalytic degradation of Methylene Blue (99.58%), Rhodamine B (100%) and Crystal Violet (98.65%).

Two nanocomposites were prepared with varying compositions of ZnO and acid-activated kaolinite for use as photocatalysts to degrade Methylene Blue [135]. The ZK-30 nanocomposite (30 wt% ZnO) removed 98% of the dye from an aqueous solution of pH 10. The recyclability experiments about the continuous use of the nanocomposites showed 80–99% dye removal for up to three consecutive photocatalytic cycles.

N/p-type nanomaterials loaded on the surface of cellulose nanoparticles had been used to fabricate hybrid nanomaterials to be utilized in the photocatalytic degradation of Methylene Blue and Rhodamine B dyes [136]. In single-dye solutions, the degradation efficiency reached 95% for Methylene Blue and 47.4% in the case of Rhodamine B dye, whereas in a mixed dye solution, the degradation reached 53% and 89.8% for Rhodamine B and Methylene Blue, respectively.

Titanium dioxide nanoparticles were prepared by a sol-gel process and calcined for 2 h at temperatures in the 300 °C–600 °C range [137]. The increase in the calcination temperature produced an increase in the size of the nanoparticles. The photodegradation performance of these nanoparticles was investigated in the removal of 10 ppm Methylene Blue from water, showing the nanomaterial calcined at 400 °C the best efficiency (near 95%) over the other nanoparticles examined in this work.

The tannic acid coating method was utilized to synthesize Ag nanoparticles-loaded ultralong hydroxyapatite (HAP). The nanowires were employed as building blocks together with chitosan (CS) to fabricate highly porous flow-through reactors (Ag@HAP/CS) for continuous catalytic reduction of Methylene Blue [138]. These nanomaterials presented efficiencies of near 99% at high fluxes, i.e., 2000 L/m^2^·h, and using a very low concentration of NaBH_4_ i.e., dye:NaBH_4_ relationship of 1.

Pseudobrookite (Fe_2_TiO_5_) was fabricated and used in the photocatalytic degradation of Rhodamine B [139]. The degradation process was associated with the adsorption of protons and the formation of an electron–hole pair. The nanomaterial thermally treated under nitrogen atmosphere presented better catalytic activity than those fabricated in air.

Reference [140] described the use of natural cotton substrates and the in situ mineralization of β-FeOOH nanorods as precursors in the synthesis of an inorganic–organic compound semiconductor nanomaterials (Cot-FeOOH). These materials had photocatalytic activity (98%) for Rhodamine B dye under visible light irradiation and in the presence of hydrogen peroxide.

Fe_3_O_4_ nanoparticles with a size of 350 ± 50 nm were synthesized by a solvothermal method. Then, the nanoparticles were coated with a shell made of 3-aminophenol-formaldehyde (APF) resin to prevent agglomeration, and further, core–shell Fe_3_O_4_@APF nanospheres were produced by polycondensation within 10 min [141]. The magnetic Fe_3_O_4_@APF@Ag nanomaterials catalyzed the reduction via the use of sodium borohydride, of Rhodamine B and Methylene Blue dyes, with conversion of these chemicals above 90% within 3 min of reaction time and a slight decrease in the reduction efficiency after seven cycles.

Ag_2_O nanoparticles, with cubic structure and crystalline diameter of 18.5 nm, were prepared and used in the photocatalytic degradation of Rhodamine B [142]. The exposure of an aqueous solution containing the dye to UV-visible light during one hour produced a 76% reduction in the compound. The main factors responsible in the degradation process were the reactions with super oxide radical (O_2_^•^) and the hydroxyl radical (^•^OH).

Nanoparticles (average size of 1.87 nm) composed of Fe_3_O_4_/Mn_3_O_4_/CuO were used as a nanocatalyst in the degradation of Methylene Blue under ultrasonic conditions [143]. The degradation of the dye was investigated under various experimental conditions, with the optimal conditions as follows: temperature of 28^°C^, 0.03 g/L initial dye concentration, 1.0 g/L nanoparticles dosage, 5 mM of H_2_O_2_, and 60 kHz. A maximum 95.04% dye degradation using these nanoparticles was reached after 150 min.

Using attapulgite (ATP) as carrier, Fe_3_O_4_ and g-C_3_N_4_ were grafted onto ATP, and the surface was then modified with polyethyleneimine (PEI) to produce photocatalyst ATP-Fe_3_O_4_-g-C_3_N_4_-PEI, which was used in the treatment of a Malachite Green-bearing wastewater [144]. Electron paramagnetic resonance analysis confirmed that the nanocomposite generated hydroxyl radicals (·OH) and superoxide radicals (·O_2_^−^), which degraded the dye. Under the action of H^+^, ·O_2_^−^, and ·OH, a removal rate of 98% was reached at pH 3. Recyclability experiments showed that there was a continuous degradation of the photocatalyst from the first (98% dye removal efficiency) to the fourth (65% dye removal efficiency) cycle.

The controlled synthesis of bismuth with various morphologies—nanoparticles (20–50 nm), nanorods (diameter of 20 nm and length 1 µm), nanocubes (150–200 nm) and micro-spheres (10 µm)—has been investigated [145]. The degradation efficiency, after 90 min of exposure to visible-light irradiation, against the presence of Rhodamine B in solutions was 99.9%, 98.9%, 97% and 68.3% for the above morphologies, respectively. The catalytic activity of the same morphologies increased with the decrease in pH value, maintaining a removal rate of 73.5% after forty cycles. Remarkably, this is a work that investigates the reusability of these adsorbents for a number of cycles as high as 40, when the ordinary number rarely exceeded five cycles.

Superparamagnetic Fe_3_O_4_ nanoparticles synthesized via an electrochemical procedure were used as a catalyst for the oxidation of Rhodamine B in a photo-Fenton-like process [146]. The rate of color removal was 85% after 12 min at dye concentration of 2 mg/L, H_2_O_2_ concentration of 0.18 mM, and Fe_3_O_4_ concentration of 0.2 g/L. In addition, electrochemically synthesized superparamagnetic Fe_3_O_4_ nanoparticles showed that the catalyst activity decreased from 85% in the first cycle to 78% in the fifth cycle.

Methylene Blue was the target to remove in the next few references. Silver chromate/reduced graphene oxide nanocomposites (Ag_2_CrO_4_/rGO NCs) with a narrow dissemination size were used for the removal of Methylene Blue [147]. The photodegradation of the dye was carried out under solar light irradiation. Experimental results indicated removal efficiencies of near 92% after one hour of irradiation, a result that compared well with the results reached by pure Ag_2_CrO_4_ (46%) and rGO (30%) nanomaterials. The nanocomposites maintained their efficiency to degrade for up to five cycles.

The spherical shaped monoclinic structures, ZnO (24.9 nm) and CuO (17.0 nm) nanoparticles and ZnO/CuO (22.6 nm) nanocomposites were synthesized using extract of *Musa acuminate* fruit peel as the stabilizing and capping agent [148]. Degradation investigations on Methylene Blue had been carried out using these nanomaterials under a visible light source. ZnO, CuO, and ZnO/CuO showed degradation efficiencies of 57%, 50%, and 90%, respectively.

Polypyrrole (Ppy)-In_2_O_3_ nanoparticles hybrids were fabricated to leverage its superoxide radical decomposition for Methylene Blue degradation [149].

The nanoemulsion procedure was utilized to synthesize Bi_x_Sn_6−2x_S_y_ (0.33 ≤ x≤ 2.95) photocatalysts with morphological structures that changed from nanowhiskers to quantum dots [150]. Particularly, BiSn_4_S_4.5_ formulation, photodegraded Methylene Blue in the shortest time under UV-visible light (Table 8).

Copper oxide (CuO) was used to dope tin oxide (SnO_2_) in order to yield a heterostructured photocatalyst, which was used in the degradation of Methylene Blue [151]. The oxide mixture degraded the dye with 90% efficiency in three hours, which improved the results derived with the use of SnO_2_.

Black titanium dioxide (BTO) nanoparticles were formed, using pulsed laser irradiation in liquids, from de-ionized water, isopropyl alcohol, and 1:1 mixtures of water and alcohol [152]. Photocatalytic degradation of Methylene Blue was improved at neutral pH values of the solution. This degradation occurred via reaction of the dye with ·OH, O_2_^·−^and ·OOH radicals to produce CO_2_ and water. The efficiency decay for every adsorbent was approximately 10% after continuous use (three cycles), though for the four adsorbents tested, only BTOWA degraded the dye at 54% in the first cycle.

With Ag-manganese oxide, with plasmon-enhanced photocatalytic activity, the photodegradation of Crystal Violet dye was formed. Manganese oxide was prepared by an acidic precipitation method using potassium permanganate, manganese acetate, and nitric acid as precursors. Silver nanoparticles were deposited on the manganese oxide using leaf extracts of *Calotropis gigantean* [153]. The deposition of silver increased the photocatalytic activity of the manganese oxide from 68 to 95%. By the use of 10% Ag-OMS, near 100, 95, and 75% efficiencies in the photodegradation of 50, 100, and 150 mg/L dye concentrations were observed in 90, 120, and 120 min, respectively. After three cycles of activity, the efficiency to degrade the dye was maintained above 90%.

Pristine and carboxylic acid (CXA)-modified MIL-53 (Al) nanostructures were used to investigate its performance on the adsorption of Rhodamine B and Methylene Blue from solutions [154]. The incorporation of the acidic function to MIL-53 (Al) produced an increase in the adsorption capacity, though Rhodamine B was adsorbed better than Methylene Blue due to its higher adsorption energy.

Cellulose acetate/polycaprolactone (CA/PCL)-based nanocomposites with manganese tungstate (MnWO_4_) nanoparticles were formed and used, under a UV light source, on Crystal Violet photocatalytic degradation [155]. The photocatalytic efficacy of the CA/PCL/MnWO_4_ nanocomposites was higher than that of the individual components.

Two-dimensionally layered molybdenum disulphide/boron nitrate/reduced graphene oxide (MoS_2_by/BN/rGO) ternary nanocomposites was formed by sonication assisted hydrothermal procedure, and its potential to treat water contaminated with Methylene Blue was investigated [156]. The nanocomposite ability to degrade the dye reached 98% after 45 min, with a ten percent loss of effectiveness, with respect to the first cycle, in the seventh cycle. The presence of superoxide radicals (·O_2_^−^) and hydroxyl radicals (·OH^−^) were responsible for the degradation process.

TiO_2_ nanotubes were annealed in the 400–900 °C temperatures range to yield different nanomaterials and marked as TiO_2_ ST-NT, moreover, part of these nanopowders were decorated with Fe_3_O_4_ nanoparticles to form TiO_2_ ST-NT@Fe_3_O_4_NPs [157]. These nanocompounds were investigated for the photocatalytic decomposition of Methylene Blue under UV light. The best photocatalytic activity of TiO_2_ ST-NT and TiO_2_ ST-NT@Fe_3_O_4_NPs was obtained at annealing temperatures of 600 and 700 °C, respectively.

Graphitic carbon nitride (g-C_3_N_4_) photocatalysts were prepared using mixtures of urea and thiourea, as precursors, by varying calcination temperatures ranging from 500 to 650 °C for three hours in air medium, and used to remove Rhodamine B from liquid solutions [158]. The results showed that Rhodamine B removal by g-C_3_N_4_ formed at 600 °C was near 95% within three hours of visible LED light irradiation

NiMn_2_O_4_ nanoparticles were prepared through a PEG-assisted hydrothermal method. This Ni4 nanosphere was used as a visible light photocatalyst for the degradation of Methylene Blue and Rhodamine B dyes [159]. Under optimized conditions and 210 min, the degradation rates of the two dyes were 68 and 80.7%, respectively, whereas under five cycles of continuous use, the efficiency was maintained above 95% for both dyes.

Zinc oxide nanoparticles with spherical (40–100 nm) and rod (200 nm) shapes were fabricated using the extract from *Scallion peel* and used to remove Methylene Blue presented in a mimic wastewater [160]. UV and visible light irradiation were used to degrade the dye. Experimental results showed that UV light was more effective (10 min) than visible light (30 min) to eliminate the dye from the solution.

Z-scheme V_2_O_5_/g-C_3_N_4_ photocatalytic composites were synthesized and used to degrade Methylene Blue [161]. This investigation showed that the optimum composite formulation had a 90% efficiency in the degradation of the dye, a result that was approximately 6.18-fold higher than that of pristine GCN catalyst. After five cycles of continuous use, the GVO2 heterostructure showed a decrease in its degradation efficiency (from 90% in the first cycle to 80% in the fifth cycle).

Zn- and Fe-co-doped TiO_2_ photocatalyst were fabricated by the sol-gel method at room temperature, presenting the tetragonal anatase phase of TiO_2_ in all synthesized nanoparticles [162]. Also, the presence of spherical nanoparticles in ZFT_2.5 photocatalyst with diameters ranging from 8 to 20 nm was shown. The photocatalyst ZFT_2.5 was investigated for the photocatalytic degradation of a mixture of three cationic dyes (Rhodamine B, Malachite Green and Methylene Blue) under exposure to visible light. Experimental results indicated that the above dyes degraded with 90.57%, 91.54% and 88.39%, respectively.

Graphite oxide and carbon quantum dots (CQDs) were added to polyacrylonitrile nanofibers to investigate its performance, under visible light irradiation, on the photocatalytic degradation of Methylene Blue. Quantitative degradation efficiency for the dye was found after 25 min, showing the nanocomposite had better efficiency than the pristine precursors [163].

A green procedure was used to create MgO NPs using *Manilkara zapota* as a bio source; further, activated carbon/magnesium oxide (AC/MgO) photocatalyst was blended through a solution evaporation procedure [164]. This photocatalyst was used for the photodegradation of Rhodamine B using a UV–visible spectrophotometer. The dye can be degraded (99%) under simulated solar irradiation.

Cadmium-doped ZnO nanocomposites was prepared to investigate its performance on the photocatalytic degradation of Methylene Blue [165]. Cadmium doping in ZnO nanostructure acted as an electron scavenger, stopping electron–hole (e^−^/h^+^) pairs from recombination on the surface of ZnO and increasing charge transfer.

Having as a target the removal of the same dye as in the previous reference, pure and TiO_2_-doped MnO_2_ spherical nanoparticles were made using a sol-gel technique. These nanoparticles’ photocatalytic degradation, under visible light exposure, of Methylene Blue was investigated [166]. However, the use of the titanium-bearing nanomaterial only produced, after 120 min, a little improvement over the use of MnO_2_. The utilization of both nanomaterials under continuous cycles indicated that there was a continuous loss of the activity after five cycles, being more evident in the case of MnO_2_ than in the TiO_2_-MnO_2_ material.

Titanium dioxide nanoparticles (spherical shape) were formed, using leaf extracts of a sausage tree (*Kigelia Africana*), and evaluated for the photocatalytic degradation of Toluidine Blue [167]. This nanomaterial removed the dye with a 99.6% efficiency within one hour and under a UV–visible source.

A MoS_2_/FeMoO_4_ composite was formed by introducing MoS_2_ (which actuates as an inorganic promoter) into MIL-53(Fe)-derived PMS-activator [168]. The prepared MoS2/FeMoO_4_ activated peroxymonosulfate (PMS) reached 99.7% of Rhodamine B degradation after 20 min. Fe(II) and sulfur vacancies were both responsible as the main active sites on catalyst surface, where sulfur vacancies can promote adsorption and electron migration between PMS and MoS_2_/FeMoO_4_ to boost peroxide bond activation.

Rough copper oxide with a nanostructural morphology and located on the surface of a copper sheet was formed by an electrochemical anodic oxidation procedure; these nanostructures were used in the degradation of Rhodamine 6G under the irradiation of visible light [169].

Silver-doped borophene and the corresponding zero-dimensional boron were constructed [170]. They were used to investigate its performance on the photocatalytic degradation, under UV–visible irradiation, of Rhodamine B. The dye completely degraded after 120 min, though a small but continuous decrease in the efficiency after the second and third cycles was shown.

The same dye (Rhodamine B) as in the previous reference was photocatalytically degraded by the action of Bi_2_O_3_@Zn-MOF hybrid nanomaterials, which were synthesized by supporting Zn-based metal-organic framework (Zn-MOF) through a hydrothermal process [171]. A catalytic efficiency of 97% was reached after 90 min of exposure to visible light irradiation. The generation of free radicals (·O_2_^−^ and ·OH) was responsible for the decomposition of the dye into CO_2_ and water. The nanocomposite could be reused three times, though the degradation efficiency decreased to 78%.

MnS_2_/MnO_2_-CC heterostructure dual-functional catalysts formed of ultrathin nanosheets were prepared by a two-step electrodeposition method for used in the degradation of Methylene Blue [172]. The dye removal efficiency of the heterostructural catalyst with a better kinetic rate (0.0226) can reach 97.76%, which is much higher than that of the MnO_x_-CC catalyst (72.10%).

Based on biopolymer pomelo peels (PP) and metal-oxide catalyst manganese oxide (MnO_x_), an adsorption-enhanced catalyst (MnO_x_-PP) was constructed for catalytic Methylene Blue oxidative degradation [173]. The dye was adsorbed onto the biopolymer, and then the continuous generation of active substances (O_2_^·^ and OH^·^) degraded it via an oxidative process of the adsorbed dye molecules. Methylene Blue was degraded at a 99.5% rate at 25°C and one hour, with a slight (5%) but continuous dynamic degradation efficiency during 72 h based on the self-built continuous single-pass dye purification device.

### 3.4. Remarks on the Use of Nanomaterials in the Removal of Cationic Dyes from Solutions

In the case of cationic dyes, the use of (photo)-catalysis methodology surpasses the utilization of adsorption–elution processes to remove these harmful dyes. A new approach, the formulation of nanomaterials bearing mixed matrix membranes, has been developed for the removal of cationic dyes (Methylene Blue) from a simulated wastewater. Like in the case of anionic dyes, it is extremely subjective, independently of the used methodology, to compare one nanomaterial to another due to the various experimental conditions used in the works. In the case of (photo)-catalytic approaches, times to degrade the very same dye vary from 10 min to 3 h (Methylene Blue) or 12 min to 3 h (Rhodamine B), whereas in the case of adsorption processing, the dye uptakes also show a variety of numbers. Table 9 shows a list of dyes used in various investigations. In the case of cationic dyes, Methylene Blue is the dye most investigated using any removal methodology. Table 10 summarizes some results about efficiencies found in the removal of Methylene Blue or Rhodamine B using the (photo)-catalytic approach; it can be seen that efficiencies in the removal of these dyes ranged from a few minutes to hours. It is also worth noticing here that despite the good removal characteristics of the different nanomaterials, very few show significant stability under continuous use. Also, none of these investigations used real wastewater.

**Table 9:** Summary of methodologies and cationic dyes used in the investigations.

Methodology	Cationic Dye	Reference
Adsorption	Crystal Violet Basic Fuchsin Golden Yellow, Methylene Blue Basic Red 114 Malachite Green Methylene Blue, Methylene Violet, Brilliant Green Toluidine Blue O Rhodamine B	[108] [109] [110,111,114,116,117,118,119] [111] [113,120] [114] [115] [118]
Membranes	Methylene Blue	[121]
(Photo)-catalysis	Methylene Blue Rhodamine B Malachite Green Crystal Violet Toluidine B Rhodamine 6G	[122,123,124,126,127,128,129,130,131,134,135,136,137,138,141,143,147,148,149,150,151,152,154,156,157,160,161,162,163,164,165,166,172,173] [125,126,127,133,134,139,140,141,142,145,146,158,159,162,165,166,170,171] [126,132,144,162] [132,134,153,155] [167] [170]

**Table 10:** (Photo)-catalytic removal of Methylene Blue and Rhodamine B.

Dye	Nanomaterial	Efficiency-Time	Reference
Methylene Blue Rhodamine B	Mn_4_(P_2_O_7_) Ce-Co,Fe ferrites La-Co,Fe,Ti composite Fe_3_O_4_@APF@Ag Mixed Fe,Mn,Cu oxides AgCrO_4_-rGO CuO-SnO_2_ NiMn_2_O_4_ CO-CQ dots Fe_3_O_4_@APF@Ag AgO nanoparticles Fe_3_O_4_ nanoparticles g-C_3_N_4_ NiMn_2_O_4_ Mo,Fe-sulfur-oxide	99%-30 min 95%-1 h 99%-50 min 90%-3 min 95%-2.5 h 92%-1 h 90%-3 h 68%-3.5 h 100%-25 min 90%-3 min 76%-1 h 85%-12 min 95%-3 h 81%-3.5 h 99.7%-20 min	[124] [127] [131] [141] [143] [147] [151] [159] [163] [141] [142] [146] [158] [159] [168]

## 4. Nanomaterials for the Removal of Anionic and Cationic Dyes

Different from the previous sections, in which nanomaterials were developed to remove anionic or cationic dyes, some scientists developed nanomaterials to be applied on the removal of both anionic and cationic dyes from solutions. This section reviews the performance of such nanomaterials with double use.

### 4.1. Adsorption of Anionic and Cationic Dyes on Nanomaterials

The performance of black liquor-based aerogels with carbon nanostructures (BL-CN) against dye uptake was compared with that of carbon nanostructure aerogels (CN) without the presence of black liquors [174]. Experimental results concluded that BL-CN aerogels presented better adsorption loadings of Methylene Blue and Brilliant Blue than those produced by CN-based aerogels: 256 mg/g (CN) versus 307 mg/g (BL-CN) for Methylene Blue, and 7 mg/g (CN) to 61 mg/g (BL-CN) for Brilliant Blue. BL-CN materials from pulping of RS with alkaline reagents (NaOH and KOH–NH_4_OH) were the basis for the best Methylene Blue adsorbents, whereas best Brilliant Blue uptakes were obtained with BL-CN adsorbents from sulfite reagent.

Next, reference [175] investigated the performance of biological graphene hydrogel (BGH) formed by *Shewanella putrefaciens* CN32 on the removal of Methyl Orange and Methylene Blue. Using BGHs and 24 h of reaction time, removal efficiencies reached near 93% and 91% for the respective anionic and cationic dye, increasing the efficiency with respect to the use of non-graphene materials. There are no data about the recyclability of the nanomaterial.

Carbon dots/silica nanoaggregates for dye adsorption were synthesized via a hydrothermal reaction of N-doped carbon dots with (3-aminopropyl)triethoxysilane. With experimental data fitted to the Langmuir isotherm model, these nanoaggregates presented adsorption uptakes of 1327 mg/g for Alizarin Red S and 4091 mg/g for Malachite Green [176].

The next reference, [177], presented a machine learning investigation about the adsorption of organic dyes onto nanozeolites to conclude which variables influenced their uptake from wastewater. Four ML algorithms (random forest, light gradient boosting, eXtreme gradient boosting, and artificial neutral network) were tested as regression models, with XGB presenting the best usefulness in terms of prediction of the adsorption uptake of nanozeolite from a relatively small dataset. The data concluded that cationic dye (Crystal Violet and Acridrine Orange) uptake (about 12 mg/g) is much better than that of anionic dyes (less than 0.5 mg/g) with these nanoadsorbents.

Cellulose was obtained from coconut husk fiber via an alkali–acid hydrolysis procedure with formation of nano-crystalline cellulose (NCC), presenting spherical shape with diameters below 40 nm [178]. NCC was investigated for its adsorption capacity against various dyes, with removal efficiencies near 91% for Methylene Blue and 90% for Congo Red at all pH values, with the adsorption of Crystal Violet reaching a maximum at pH 9, and Methyl Red at pH 5.

*Spirulina*-mediated titanium oxide nanoparticles (STONPs) were fabricated and used to investigate their performance on the removal of Methyl Orange and Malachite Green [179]. Methyl Orange was best removed from the solution at pH 4, whereas the pH value for maximum Malachite Green removal was 12. The maximum uptake using the Langmuir isotherm model was 272.5 mg/g and 209.6 mg/g for the respective dyes. There was a continuous and important loss of removal efficiency after five cycles

### 4.2. Nanomaterials—Forming Membranes for the Removal of Anionic and Cationic Dyes

As one can see from the next references, the use of these membranes on the removal of solutions containing anionic and cationic dyes is finding widespread use in comparison with the use of these membranes in the case of single cationic or anionic dye-bearing solutions.

An MXene nanocomposite functionalized with a zwitterion (Z-MXene) was fabricated to be used as nanofiller in the development of self-cleaning permeable membranes [180]. The presence of functional groups and negative charge on the nanocomposite surface improved the separation of Congo Red and Methylene Blue by near 275 and 400%, respectively; at the same time, a high water flux was maintained. The composite membrane is chemically stable in the presence of corrosive (2 M HCl) and oxidative (NaOCl) media.

A polyacrylonitrile (PAN) electrospun membrane was constructed as a scaffold to incorporate nanomaterial adsorbents in a layer-by-layer hierarchical structure [181]. Two different nanomaterials were incorporated: a positively charged metal–organic framework as MIL-101(Cr), and negatively charged functionalized multi-walled carbon nanotubes; these were used to remove anionic (Methyl Orange and Rose Bengal) and cationic (Methylene Blue) dyes. Operating at 0.04 bar, removal efficiencies of the membrane incorporating MIL-101(Cr) were 5% (Methyl Orange) and 99% (Rose Bengal) from an iso-propylic alcohol medium. Multiwalled carbon nanotubes (MWCNTs)-based membrane showed 90% removal of Methylene Blue also in the same alcoholic medium, and operating at 0.07 bar.

Graphene oxide–copper oxide (GO–CuO) nanomaterial was incorporated into cellulose-acetate (CA) and poly-ether sulfone (PES) blend polymer by using a phase-inversion process to fabricate thin-film nanocomposite (TFN) membranes, which were used on the removal of Methylene Blue, Rhodamine B, Methyl Orange and Congo Red [182]. In equilibrium conditions, the composite membrane removed 92%, 89%, 59% and 68% of the above respective dyes; thus, the TFN membrane seemed to be more effective to remove cationic dyes than anionic ones. The membrane was also used on the purification of a textile industry effluent sample (with no mention of the dyes contained in it), and all physico-chemical properties of the sample showed a decrease in their values when compared to the respective ones of the laboratory synthetic effluent.

Heterojunctions formed by UiO-66-NH_2_ and carbon quantum dots (CQDs) to investigate their synergetic advantages as nanofillers in thin-film nanocomposite (TFN) membranes for dye removal were utilized [183]. Compared to the pristine thin-film composite membranes, the optimal TFN membranes embedded with 0.2 wt% UiO-66-NH_2_/CQD improved pure water permeability by 89.7% and higher rejection of dyes of different charges and molecular weights (Methylene Blue, Methyl Blue, Methyl Orange and Direct Red 23). Moreover, the decrease in water flux caused by dye adsorption during the long-term filtration process can be recovered by 92–99% via photocatalytic degradation of the embedded dyes within 10 min.

### 4.3. (Photo)-Catalytic Degradation of Anionic and Cationic Dyes Using Nanomaterials

A mesoporous rod-shaped ZnO/CuO/CeO_2_ n-p-n heterojunction via a two-step co-precipitation technique was formed to investigate its use in the degradation of Crystal Violet and Methyl Orange [184]. Under sunlight, the degradation efficiencies were 96% (Crystal Violet) and 88% (Methyl Orange) after 90 min of irradiation. The ultimate oxidizing species that degraded the dyes were O_2_^−^ and •OH over photocatalyst under sunlight illumination. Also, the recycling experiments showed that the nanomaterial maintained its activity after three cycles.

Lanthanum- and neodymium-substituted cobalt–strontium (Co–Sr) spinel ferrite (Co_0.5_Sr_0.5_RE_x_Fe_2-x_O_4_, x = 0.00 and 0.06) catalysts were synthesized and used to degrade Congo Red and Rhodamine B dyes from an aqueous solution mixture [185]. It was determined that the energy bandgap ranged from 2.91 to 2.52 eV. The formulation in which x= 0.06 presented the greatest degradation rates for both dyes, being the ferrite containing Nd^3+^, which was the species that presented the highest rates of all (Table 11).

α-Fe_2_O_3_ was built at the nanometer (nanofibers) level via an electrospinning procedure to yield a nanomaterial with advanced characteristics to be used as photocatalysts to degrade Rhodamine B and Methyl Orange dyes [186]. The nanofibers fabricated from FeCl_3_·6H_2_O presented the best photocatalytic degradation, under visible-light irradiation, against the presence of both dyes in solution. The iron source is key to yielding a photocatalyst with the best degradation properties.

A solvothermal method was used to form mesoporous TiO_2_, and (1–3 wt%) Cu-doped mesoporous TiO_2_ membrane. Using artificial light source exposure, the effectiveness of the above materials on the degradation of Congo Red and Methylene Blue was investigated [187]. Cu-doping shifted the light absorption of mesoporous TiO_2_ from the ultraviolet to the visible region. Additionally, 3 wt% Cu-doped mesoporousTiO_2_ photocatalyst showed lower band gap energy (2.6 eV) than TiO_2_ (3.2 eV), thus allowing the use of solar energy, and the exposure was shifted towards the visible region. Congo Red was degraded a 61% using mesoporous TiO_2_ material, whereas the degradation reached 99% under the use of 3 wt% Cu-doped mesoporous TiO_2_ material and pH of 5. In both cases, the light exposure time was 50 min. In the case of Methylene Blue, maximum degradation (95%) occurred at a pH value of 9.

In [188], the usage of Au/WO_3_ composite as a photocatalyst for the degradation of dyes under solar light irradiation was investigated. Au/WO_3_ nanocomposites (nanoplatelets and pseudospheres) were synthesized using an acid precipitation method followed by an impregnation/reduction at room temperature. The derived composites were formed with gold nanoparticles of 7 nm in size, whereas W(VI) was reduced to the W(V) oxidation state, and favoring the presence of oxygen vacancies and the presence of a surface plasmon resonance effect at 540 nm. At pH 5, the degradation produced with these Au/WO_3_ photocatalysts was highly efficient on cationic dyes (Methylene Blue and Rhodamine B) rather than in Methyl Orange (anionic dye). Au/WO_3_-forming pseudosphere was the most efficient degrading material of cationic dyes (near quantitative degradation at 60 min and 90 min for Rhodamine B and Methylene Blue, respectively). In the case of Methyl Orange, the gold–tungsten oxide was useless at alkaline pH values at degrading the dye, but at acidic pH values, the degradation of the dye reached 80%.

Chitosan-capped silver nanoparticle (Ag NPs-CS) adsorbents to remediate dye pollution were photochemically formed under direct sunlight [189]. An aqueous Ag NPs-CS (100 μg/mL) degraded more than 95% of a mixed dye solution (25 mg/mL) containing equal volumes of Rhodamine B, Methylene Blue and Methyl Orange.

ZnO and Ag/ZnO nanocomposites were fabricated from *Basella alba* aqueous leaf extract. The Ag/ZnO nanocomposite showed degradation properties against the presence of Rhodamine B, Methylene Blue, and Methyl Orange, with degradation efficiencies of 88%, 90%, and 92% for the respective dyes [190].

Copper-based organic–inorganic hybrid nanoflowers (Cu-hNFs) were obtained from *Tornabea scutellifera* lichen extract as the organic component [191]. Catalytic activity was shown against Methylene Blue (maximum degradation effectiveness at pH 7.4) and Brillant Blue (best removed at pH 5). Both dyes degraded via a Fenton-like process.

α-MoO_3_ nanoparticles synthesized by chemical co-precipitation were used as photocatalysts on the degradation of Methyl Orange and Methylene Blue under visible light exposure. Maximum degradation efficiencies reached 73% (anionic dye) and 95% (cationic dye) after 90 min [192].

Three commercially available ZnO powders were used for the tribocatalytic degradation of Rhodamine B, Methyl Violet, Methyl Blue and Methyl Orange dyes [193]. The removal of Rhodamine B reached near 99% efficiency after two hours, whereas after five hours of reaction time, the catalytic efficiencies in the removal of the separate dyes, followed the next order: Methyl Violet (99%) > Methyl Orange (85%) > Methyl Blue (55%). In the process, h+, ·O_2_^−^ and ·OH were generated, these radicals being responsible for the degradation of the respective dyes.

Bimetallic oxides were formed after the calcination of nanomaterials derived from metal–organic frameworks. When Mn(M)-BTC nanomaterials were doped with Cu^2+^, Fe^2+^, Ce^3+^ or trimesic acid (H_3_BTC), several derivatives were formed, including material containing Cu^2+^, the nanomaterial that presented the best degradation efficiency of several dyes: Methylene Blue, Rhodamine 6G, Eosin Y and Gentian Violet. In all these cases, the degradation efficiency was above 95% [194].

Semiconductor heterojunction systems of C-modified Zn-doped TiO_2_ composite nanomaterials with nanofiber structures (150–200 nm diameter) were synthesized by electrospinning and hydrothermal methods [195]. Against the presence of Methylene Blue, Methyl Orange, Rhodamine B and Malachite Green, the composite nanofiber film had photocatalytic activity for all dyes attributable to the large specific surface area, small size effect and synergistic effects of multiple heterojunctions and dopant atom. The removal efficiency followed the Malachite Green > Methylene Blue > Methyl Orange > Rhodamine B order, with Methyl Orange efficiently removed in just 15 min.

Hybrid multifunctional nanoplexes composed of ZnS semiconductor quantum dots (ZnS QDs) chemically biofunctionalized with ε-poly-L-lysine (ɛPL) and coupled with magnetic iron oxide nanoparticles (MION, Fe_3_O_4_) stabilized by carboxymethylcellulose (CMC) were fabricated [196]. These nanoplexes were used to investigate its photocatalytic activity in the presence of Methylene Blue, Methyl Orange, Congo Red, and Rhodamine dyes. Degradation occurred via oxidation of the dyes by ·O_2_^−^and ·OH radicals, and also by the oxidation of dyes by holes:

(1) $$ZnSQD{(h}_{VB}^{+})+dye\to degradationby-products$$

In this investigation [197], ZnO nanoparticles (average size 15–30 nm) were synthesized by biosynthesis using dried *Peumus boldus* leaves. The photocatalytic activity of these nanomaterials indicated that the quantitative degradation of Methylene Blue is possible in three hours, 92% in the case of Methyl Orange in the same time, and 100% degradation of Rhodamine B in 30 min.

n-type semiconductor ZnO was coupled to a p-type semiconductor CuO to form a p–n heterojunction. Further, the active surface of the heterojunction was increased by the addition of a SiO_2_-aerogel [198]. By the application of ultrasound, aerogel particles were better distributed, and at the same time, the number of active sites was increased. CuO-ZnO/SA(P= 200) formulation (1 g/L) photocatalytically degraded 20 mg/L of each dye at pH 8: 95.4% in the case of Methylene Blue, whereas Methyl Orange degraded by 56.1% and Congo Red by 71.3%. In the case of Methylene Blue, there was a continuous loss of efficiency after four cycles: 90.6% in the first cycle versus 78% in the fourth one.

Several materials, such as graphene oxide, magnetite-(FeO·Fe_2_O_3_), and hematite-(α-Fe_2_O_3_)-decorated graphene oxide, were investigated as integrated photocatalyst adsorbents (IPCAs) in the oxidation of Methylene Blue, Crystal Violet and Tartrazine Yellow [199]. The cationic dyes showed maximum degradation, whereas the anionic dye presented acceptable removal rates when iron-decorated nanomaterials were used. In these cases, the efficiency order was as follows: graphene oxide-αFe_2_O_3_>graphene oxide-Fe_3_O_4_>graphene oxide. All the nanomaterials maintained their photocatalytic activity for five cycles, with dye removal in the 86–95% range.

Zinc selenide nanomaterials, produced from waste peels of orange and potato using the hydrothermal method, were utilized as photocatalysts to degrade Methylene Blue and Congo Red using sunlight [200]. Citrate in orange peel-mediated synthesis helped to form particle sizes of 1.85 nm and a surface area of 17.078 m^2^/g, resulting in an increase in surface-active sites. These nanomaterials had a degradation efficiency of 97.16% and 93.61% for Methylene Blue and Congo Red, respectively.

Magnetic SiO_2_ (MagSiO_2_@PDA@MnO_2_) were synthesized using magnetic, monodisperse-porous SiO_2_ (MagSiO_2)_ as the starting material [201]. MagSiO_2_@PDA@MnO_2_ material was used to remove Rhodamine B, Methyl Orange and Methylene Blue, with the removal of the dyes attributable both to physical adsorption and degradation via peroxymonosulfate activation. Removal efficiency had the following sequence: Ethylene Blue>Rhodamine B>Methyl Orange sequence. The dye removal rate showed that the degradation occurred via physical adsorption and peroxymonosulfate oxidation. After five cycles, there was a small decrease in the dye removal efficiencies, probably due a decrease in the manganese content of the material.

Silver nanoparticles (AgNPs) were synthesized from *Echinophora platyloba* plant extract, which acted as both a reducing agent and capping agent in the synthesis of the nanoparticles [202]. It was found that pH played a key role in determining particle sizes, which can be tuned from 15 nm to 53 nm when the pH decreased from 10 to 7. AgNPs (15 nm) showed remarkable catalytic performance in the degradation of different individual azo dyes (i.e., Methylene Blue (90.1%), Congo Red (80.1%), Rhodamine B (87.2%), Methyl Red (80.3%), Bromocresol Blue (92.5%) and Imido Black (79.8%)), as well as their mixture. It was found that 79.2% of the mixture of dyes was decomposed in eleven minutes, following a pseudo-first order kinetic model.

A sunlight-active n–n heterojunction of TiO_2_-coupled-CdS (TiO_2_-CdS) was prepared using a seed extract of *Sapindus mukorossi* and investigated in the removal of Rhodamine B and Congo Red [203]. The nanocomposite presented degradations of 93% and 95% for the respective dyes under the following experimental conditions: 25 mg/L dyes concentration, 15 mg of nanocomposite dosage, neutral pH and five hours of reaction time. Under continuous use (eight cycles of dye uptake and degradation), there was a continuous but slight efficiency loss in the treatment of both dyes, better displayed in the case of Rhodamine B than in Congo Red.

A solvothermal procedure converted electrical furnace slag into a steel slag nanocomposite (average particle size 34.8 nm), which were supported by H_2_O_2_ for Fenton and photo-Fenton-like degradation of various dyes in both suspension and spin-coated modes [204]. Using Methylene Blue and based on the optimum dye removal conditions (30 mg/L dye concentration, pH 11, 1.59 mL/L H_2_O_2_ concentration, 58.1 mg/L nanocomposite dosage, and one hour), five cycles were carried out for suspension and spin-coated modes. In the fifth cycle, resulting data showed that 92.89% of dye degraded for suspension mode and 88.21% for spin-coated mode. At pH 7 and 30 mg/L of each dye, the photodegradation efficiency of Methylene Blue, Methyl Red, Rhodamine B, Congo Red, Acid Blue 25, and Methyl Orange depended largely on the type of dye, with cationic dyes removed more efficiently than anionic ones (Table 12).

Different heterogeneous photocatalysts composed of highly reduced graphene oxide (HRG) and vanadium oxide (VO_x_)-based nanocomposites (HRG-VOx) were formed (nanorods, nanosheets and urchins), and their photocatalytic activities to photodegrade Methylene Blue and Methyl Orange were investigated [205]. Among all the morphologies, nanocomposites consisting of urchin-shaped VO_x_ nanoparticles (HRG-VO_x_-U) demonstrated superior photocatalytic properties towards the degradation of the dyes (99.1% for Methylene Blue after 45 min, and 97.2% for Methyl Orange after 35 min). After three cycles and under UV-light irradiation, there was a continuous loss in the efficiency of Methylene Blue degradation.

A supramolecular material (3–4 nm average particle diameter) formed by the hybridization of hydrophilic hydrazide-pillar[12]arene, as surface-modifier, and silver nanoparticles was used in the reduction of Methylene Blue, Rhodamine B, Rhodamine 6G, and Methyl Orange [206]. After five cycles of continuous use, the degradation efficiency was maintained almost constant in the case of Rhodamine B and Methyl Orange dyes, but suffered a continuous loss in the case of Rhodamine 6G (most notably) and Methylene Blue.

ZnO nanoparticles (10 nm), as a photocatalyst material, were used to degrade Remazol Brilliant Blue R and Brilliant Green [207]. Zinc acetate, sodium hydroxide, polyvinylpyrrolidone and ethylene glycol at 197 °C formed the nanoparticles. After 90 min of reaction time, 500 ppm of ZnO degraded 73% and 51% of the respective dyes.

MgO–ZnO (MZ) nanocomposite (nanoplates with 35–100 nm of thickness) was prepared by a co-precipitation procedure with enhanced photodegradation properties under natural sunlight exposure [208]. Both cationic and anionic dyes were used in the degradation experiments, and it was found that both types of dyes completely degraded after 20 min of sunlight exposure. After five cycles of use, Rhodamine B, Methyl Orange, and Methylene Blue presented variations in their degradation efficiency: 96–91%, 91–85% and 98–92%, respectively.

Another nanomaterial with photodegradation properties under sunlight exposure, to degrade cationic and anionic dyes, was used in the next investigation [209]. In this case, the material was a ZnO/CuO nanocomposite with ZnO and CuO nanoparticles, and the cationic dyes were Methylene Blue and Rhodamine B, whereas Methyl Orange was the anionic dye used in the investigation. ZnO and CuO nanoparticles were synthesized by a biosynthesis procedure using *Ficus benghalensis* leaf extract, whereas the nanocomposite was fabricated by the mortar pestle crushing/milling method. As was somewhat expected, the efficiency of the nanocomposite in degrading the three dyes was higher than that yielded by the use of the single oxides. Using the nanocomposite for three hours and under sunlight exposure, Methylene Blue degraded by about 99%, compared to 80% forf Rhodamine B and 67% for Methyl Orange.

Potato-on-rod-like Z-scheme plasmon Ag_2_CrO_4_–Ag_2_Mo_2_O_7_ heterojunction nano-photocatalyst was synthesized by a precipitation method to photodegrade different organic dyes under artificial sunlight [210]. Under the best experimental conditions of a 0.1 g dose of (3:1) nanophotocatalyst, 10 mg/L dye concentration and 90 min of exposure to light, the above nanophotocatalyst formulation exhibited the greatest efficiency in the photodegradation of 2-Naphthol Orange (97.8%), Rhodamine B (99.7%), Crystal Violet (98.9%), and Methyl Orange (56.1%). In the case of 2-Naphtol Orange, the 3:1 catalyst formulation showed an acceptable reusability, since the dye adsorption ranged from 97.8% (1st cycle) to 95.7% in the fourth cycle.

A one-step hydrothermal procedure was used to form Bi_2_O_2.33_//Bi_2_O_3_@ECNF (electrospun carbon nanofibers), which combines both adsorption and photocatalytic performance. The nanocomposite removed 95% of Methyl Orange and 83% of Rhodamine B after 2 and 3 h, respectively. Both h^+^ and ·OH were the main active species in the photocatalytic process under light exposure [211].

### 4.4. Remarks on the Use of Nanomaterials in the Removal of Anionic and Cationic Dyes from Solutions

At first glance, the uses of these nanomaterials have an advantage over the utilization of nanomaterials with a single use in that they have a broad scope, since they are useful in the removal of anionic as well as cationic dyes from solutions. However, they present somewhat the same negative characteristics as the others, especially regarding their stability under continuous cycles, according to the data shown in the published manuscripts, because in many of the reviewed references, the authors did not mention this issue regarding their respective investigated nanomaterial. As in previous cases, comparison between the different nanomaterials is subjective due to the different experimental conditions, and in this case, another variable must be considered: the different mixtures of dyes used in the investigations. As was previously performed, Table 13 summarizes the methodologies used to remove the mixture of anionic and cationic dyes.

As an example of what it is mentioned above, using the same mixture (Methylene Blue, Rhodamine B and Methyl Orange) of dyes, the results in [208] indicate that complete degradation of the dyes occurred after 20 min, against efficiencies of 99% (Methylene Blue), 80% (Rhodamine B) or 67% (Methyl Orange) in 3 h declared in [202]. These last results hardly compared with data from [197], in which it is demonstrated that Rhodamine B was completely eliminated after 30 min. Another mixture of dyes (Methylene Blue and Methyl Orange) shows the same disparity in results, since 90 min is needed to remove 95% and 75% of the respective cationic and anionic dye [192], and 45 min (99% Methylene Blue) or 35 min (97% Methyl Orange) as concluded in [205].

## 5. Conclusions

This review emphasized the importance that the removal of organic dyes from solutions has in the scientific community; in this removal, two approaches are followed—adsorption or catalytic-photocatalytic degradation of the dye—while the use of nanomaterials also has widespread use.

In the case of adsorption methodology, all the references reviewed but two fitted its experimental data to the Langmuir isotherm model; the two exceptions are references [113,120], which fitted their data to the Freundlich isotherm. However, these nanomaterials performed quite differently in the removal of both cationic and anionic dyes, and of the very same dye, as the results in Table 14 demonstrate in the case of the adsorption of Congo Red and Methylene Blue. In the case of anionic dyes, maximum loadings vary from a few mg/g to the g/g magnitude order, and in the case of Methylene Blue, the tendency is the same. These variations can be generalized to other dyes.

With these results, it is amazing that some authors expressed their data as 8300 mg/g instead of the more logical 8.3 g/g form.

In the utilization of (photo)-catalytic methodology, the results have the same tendency as those shown in Table 14 and commented on in Section 2.3 (Table 5), Section 3.4 (Table 10) and in the text of Section 4.4. The efficient removal of a given anionic or cationic dye is reached after minutes or several hours depending of the nanomaterial used. In the opinion of the present authors, other considerations (see below) may be taking into account prior selection of one or another nanomaterial for each specific use or the methodology used to carry out this removal.

However, there is no systematic information about how the adsorption of dye molecules are related to the surface properties of nanomaterials, such as porosity, surface characteristics, etc.

A point to consider is the tendency that some authors provided data with *little* scientific sense, as is exemplified with the values of the thermodynamic data calculated from selected references (Table 15).

As is seen, values are given with three to four decimal places, which does not correspond with the accuracy of the methodology used to calculate such numbers; the responsibility for publishing such values is not only of the authors but also of the respective reviewers (and Editors) who allow this.

Since the removal of dyes from solutions is of utmost interest, future trends may be focused on the following: (i) the development of more stable nanomaterials to be used after cycles (a general rule observed in this review is that there is a loss of efficiency with continuous cycles). (ii) Investigations of the use of nanomaterials on real dye-bearing wastes (in this review, just only two, [69,182], references used industrial wastewater in their investigation). (iii) The use of dynamic conditions (columns) in the removal of dyes. (iv) Reliable knowledge about the easiness and costs of fabricating nanomaterials on greater scales than the laboratory one. All the above will be key points in understanding the performance of nanomaterials in the selected technology under long-term use.

## Figures and Tables

**Table 2:** Percentage of reusability of SONO-CFL/CN nanomaterial.

Cycle	Adsorption	Sono-Sorption
1st 2nd 3rd 4th	78.4 60.4 59.9 57.4	96.2 87.6 76.5 74.7

Experimental conditions: 0.02 g adsorbent/50 mL solution. Time: 30 min. Temperature: 25 °C. Initial dye concentration: 20 mg/L. pH: 2.0 ± 0–5. From [64].

**Table 3:** Dye uptake (mg/g) using zinc or copper ferrites in nanoparticulated form.

Nanoparticle	Alizarin Red S	Thiazol Yellog G	Congo Red	Methyl Orange
ZnFe_2_O_4_ CuFe_2_O_4_	54.6 46.4	37.0 30.1	29.8 21.9	26.8 20.8

From [69].

**Table 4:** Summary of the use of nanomaterials in the removal of anionic dyes.

Methodology	Anionic Dye	Reference
Adsorption	Acid Yellow 36, Acid Orange 6 Reactive Red 21, Reactive Blue 19 Congo Red Alizarin Red S Reactive Yellow 145, Reactive Red 195 ^a^ Various (industrial wastewater) Methyl Orange Congo Red, Brilliant Blue G-250	[60] [60] [61,63,106] [62,64,65,67,68,71,72,73,74] [66] [69] [70] [74]
(Photo)-catalysis	Congo Red, Alizarin Red S Methyl Orange Brilliant Blue Reactive Red, Orange II Orange G Congo Red,Methyl Red Reactive Black 5 AR88 Napthol Blue Black Auramine O, Xylenol Orange Tripan Blue Red 195	[74,75,78,82,83,84,90,91,94,99,101] [77,78,79,81,82,85,86,87,92,93,96,97,98,102,105] [80] [88] [89] [90] [95] [100] [103] [104] [106] [107]

^a^ Alizarin Yellow R, Thiazole Yellow G, Congo Red, and Methyl Orange.

**Table 5:** Some efficiencies in the removal of anionic dyes using (photo)-catalysis.

Dye	Nanomaterial	Efficiency/Time	Reference
Congo Red Methyl Orange Reactive Red Orange II Tripan Blue	Cu oxide nanoparticles ZnO-CuFe_2_O_4_-CNTs Eu-TiO_2_ N-Ti_3_C_2_MXene Y_2_O_3_-SiO_2_ Various nanomaterials Carbon dots Biochar-BiOCl Ag-Fe_2_O_3_ Ag-Fe_2_O_3_ Cd,Ti oxides-Ionic liquid	93%/2 h 82%/2 h 97%/75 min 98%/20 min 52%/45 min 67%/3 h 98%/7 h 100%/1 h 98%/2 h 98%/2 h 100%/1 h	[75] [76] [99] [81] [87] [95] [98] [105] [88] [88] [106]

**Table 6:** Influence of the calcination temperature on Basic Fuchsin uptake.

Temperature, °C	Dye Uptake, mg/g
No calcination 500 700	239.8 178.4 93.2

From [109].

**Table 7:** Efficiency in the removal of Methylene Blue, Methylene Violet and Brilliant Green, after 24 h, with different FePt/h-BN heteromaterials.

Sample	Methylene Blue	Methylene Violet	Brilliant Green
Boron nitride as-synthesized FPB FPB annealed at 500 °C FPB annealed at 700 °C	100 74.3 77.5 66.4	100 80 71.7 79.9	41.7 100 100 100

From [114].

**Table 8:** Percentage of degradation, under UV-visible light, of Methylene Blue using various Bi_x_Sn_6-2x_S_y_ formulations.

Material	0 h	10 min	0.25 h	1 h	3 h
SnS Bi_0.3_Sn_5.34_S_5.8_ BiSn_4_S_5.5_	0 0 0	no data no data 100	68 43 100	86 50 100	93 82 100

From [150].

**Table 11:** Percentages of dyes degradation using various Co_0.5_Sr_0.5_RE_x_Fe_2-x_O_4_ formulations.

Nanomaterial	Congo Red	Rhodamine B
Co_0.5_Sr_0.5_Fe_2_O_4_ Co_0.5_Sr_0.5_La_0.06_Fe_1.94_O_4_ Co_0.5_Sr_0.5_Nd_0.06_Fe_1.94_O_4_	73 81 90	45 67 85

Time: One hour. From [185].

**Table 12:** Efficiency in the removal of dyes with the steel slag nanocomposite.

Dye	% Efficiency
Methylene Blue Methyl Red Rhodamine B Congo Red Acid Blue 25 Methyl Orange	85.8 74.5 70.5 58.7 45.2 40.4

Time: one hour. Neutral pH. From [204].

**Table 13:** Summary of methodologies and anionic and cationic dyes used in the investigations.

Methodology	Dyes	Reference
Adsorption	Methylene Blue, Brilliant Blue Methyl Orange, Methylene Blue Alizarin Red S, Malachite Green Crystal Violet, Acridine Orange MB, CR, CV, MR Methyl Orange, Malachite Green	[174] [175] [176] [177] [178] [179]
Membranes	Congo Red, Methylene Blue MO, RB, MB MB, RhB, MO, CR MB, MeB, MO	[180] [181] [182] [183]
(Photo)-catalysis	Crystal Violet, Methyl Orange Congo Red, Rhodamine B Rhodamine B, Methyl Orange Congo Red, Methylene Blue MB, RhB, MO Methylene Blue, Methyl Orange Methylene Blue, Brilliant Blue RhB, MV, MeB, MO MB, RhB, EY, GV MB, MO, RhB, MG MB, MO, CR, RhB MB, MO, CR MB, CV, TY MB, CR, RhB, MeR MB, MeR, RhB, CR, AB25, MO MB, RhB, Rh6G, MO RBBR, BG NO, RhB, CV, MO Methyl Orange, Rhodamine B	[184] [185,203] [186] [187,200] [188,189,197,201,208,209] [190,192,205] [191] [193] [194] [195] [196] [198] [199] [202] [204] [206] [207] [210] [211]

AB25: Acid Blue 25. BG: Brilliant Green. CR: Congo Red. CV: Crystal Violet. EY: Eosin Y. GV: Gentian Violet. MB: Methylene Blue. MeB: Methyl Blue. MeR: Methyl Red. MG: Malachite Green. MO: Methyl Orange MR: Methyl Red. MV. Methyl Violet. NO: Napthol Orange. RBBR: Ramazol Brillian Blue R. RhB: Rhodamine B. Rh6G: Rhodamine 6G. RS: Rose Bengal. TY: Tartarzine yellow.

**Table 14:** Some maximum capacities of dyes on nanomaterials.

Congo Red, mg/g	Reference	Methylene Blue, mg/g	Reference
17.8 47.4 57.8 3017 4334 8300	[67] [62] [68] [71] [74] [73]	5.78 36.4 332 477 736	[129] [177] [116] [119] [112]

**Table 15:** Some strange thermodynamic values taken from the literature.

ΔH◦, kJ/mol	ΔS◦	Reference
−3.1689 54.996 3.568 7.09731	−0.0112 J/K·mol 0.283 kJ/K·mol 0.0278 kJ/K·mol 40.4502 J/K·mol	[112] [116] [119] [179]

## Supplementary Materials

The following supporting information can be downloaded at: https://www.mdpi.com/article/10.3390/ijms25179671/s1.
