# Supplementary material for: Removal of Hazardous Organic Dyes from Liquid Wastes Using Advanced Nanomaterials
Source: Int J Mol Sci. 2024 Sep 6;25(17):9671. doi: 10.3390/ijms25179671 (PMC11396100; doi:10.3390/ijms25179671)
Supplement: Supplementary file 1 [file ijms-25-09671-s001.zip › ijms-3168186-supplementary.pdf]

Removal of Hazardous Organic Dyes from Liquid Wastes Using Advanced Nanomaterials

Francisco Jose Alguacil, Manuel Alonso and Jose Ignacio Robla

Supplementary File

1. List of reviewed dyes and references

1.1. Anionic dyes

Alizarin Red S [64] [75] [82] [176]

Alizarin Yellow R [69]

AR88 [100]

Acid Orange 6 [60]

Acid Yellow 36 [60]

Acid Blue 25 [204]

Auramine O [104]

Brilliant Blue [174] [191][207]

Bromophenol Blue [80]

Bromocresol Blue [202]

Eosin Y [194]

Congo Red [62] [65] [67] [68] [69] [71] [72] [73] [74] [75] [76] [78] [83][84] [90] [91] [94][99][101] [178] [180][182] [185] [187] [196] [198] [200] [202] [203] [204]

Coomassie Brilliant Blue G-250 [74]

Direct Red 23 [183]

Methyl Blue [183]

Methyl Orange [69] [70] [77] [78] [79] [81] [85] [86] [87] [92] [93] [96] [97] [98] [102][105] [175] [179] [181] [182] [183] [184] [186] [188] [189] [190] [192] [193] [195] [196] [197] [198] [201] [204] [205] [206] [208] [209] [210][211]

Methyl Red [90][178][202] [204]

Methyl Violet [193]

2-Naphthol Orange [210]

Naphtol Blue Black [103]

Orange II [88]

Orange G [89]

Reactive Red [88]

Reactive Red 21 [61] [63]

Reactive Black 5 [95]

Reactive Blue 19 [61] [66]

Reactive Yellow 145 [66]

Reactive Red 195 [66] [107]

Rose Bengal [181]

Tartrazine Yellow [199]

Thiazole Yellow G [69]

Tripan Blue [106]

Xylenol Orange [104]

1.2. Cationic dyes

Acridrine Red [177]

Basic Fuchsin [109]

Basic Red-46 [111]

Brilliant Green [114][207]

Crystal Violet [108][132][134][153][155][177][178][184][199][210]

Gentian Violet [194]

Golden Yellow [110]

Imido Black [202]

Methylene Blue [110] [112] [114] [116] [117] [118] [119] [121][122][123][124][126][127]128][129][130][131][134][135][136][137] [138][141][143][147][148][149][150][151][152][154][156][157][159] [160][161][162][163][165][166][167][172][173][174][175][178][180][181][182][183][187][188][189][190][191][192][193][194][195][196][197][198][199][200][201][202][204][205][206][208][209]

Malachite Green [113][120][128][132][144][162][176][179][195]

Methylene Violet [114]

Rhodamine 6G [169][194][206]

Rhodamine B [125] [126][129][133] [134] [136] [139] [140] [141] [142][145][146][154][158][159][162][164][168][170][171][182][185][186][188][189][190][193][195][196][197][201][202][203][204][206][208][209][210][211]

Toluidine Blue O [115]

2. Further remarks on adsorption

The ionic characters on the surface of the adsorbent and the dye in the bulk liquid medium determine the extent of the surface binding process. The majority of the organic pollutants which are of either polar or non-polar are subjected to the electrostatic forces exerted by the adsorbents and further interactions can be mediated through physical (physisorption) or chemical forces (chemisorption).

3. Further remarks on (photo)catalytic degradation

Electronically excited photocatalysts interact with organic dyes via three main mechanisms: i) electron transfer (ET), ii) EnT, and iii) atom transfer [1].

Whereas it is described that semiconductor photocatalytic degradation process includes the following steps [2]:

i) the light irradiation induces the separation of electrons from the valence band to the conduction band and leaving the holes in the valence band,

ii) the electrons and holes migrate to the surface of catalysts and react with the electron donors or electron acceptors directly,

iii) the holes will react with surface adsorbed water molecules, forming the highly reactive hydroxyl radicals (•OH),

iv) the electrons react with molecular oxygen and produce superoxide radical ($O_2^{\bullet -}$). The radicals can further react with dyes.

Another approach is given in [3], indicating that the mechanisms of the surface-driven process can be seen as series of the next steps:

i) dye diffuses into the surface of the photocatalyst,

ii) surface adsorption,

iii) probable reactive sites/hotspots on the surface,

iv) desorption of product from the photocatalytic surface,

v) diffusion of product from the photocatalyst surface.

4. Conclusions (Prospects)

Further to what it is mentioned in the Conclusion Section, some thoughts must be done in terms about that most of the research are reporting the data only on the removal efficiency, adsorptive capacity, and the possible regeneration potential whereas information on the safe disposal of the contaminants-bounded nanomaterials find very limited and thus more data are needed in their effective clearance. Also it is important to gain further knowledge about how the adsorption of dye molecules is linked to the surface characteristics of nanomaterials, such as porosity, surface charge, etc. It is also of the utmost importance to have generic guidelines across the authorities, industries, academia and research institutions to specify the use of these nanomaterials and the probable risk associated with them (not all the world associated to these nanocompunds is as *green* as it appears at a first instance). Real-time monitoring, material toxicity, risk analysis, mechanism elucidation, the potential to reuse, fouling issues, continuous flow operations, lack of commercialization, and pursuit of new-generation versatile nanomaterials, etc. can be considered as new opportunities for scientific researcher. It must be not to forget that very often there is a mismatch between what the scientific community offers and what the industry needs.

References

[1] Amos, S.G.E; Garreau, M.; Buzzetti, L.; Waser, J. Photocatalysis with organic dyes: facile access to reactive intermediates for synthesis. *Beilstein J. Org. Chem.* **2020,** *16,* 1163–1187. DOI: 10.3762/bjoc.16.103

[2]Tong, H.; Ouyang, S.; Bi, Y.; Umezawa N.; Oshikiri, M.; Ye, J. Nano-photocatalytic materials: possibilities and challenges. *Adv. Mater.* **2012,** *24,* 229-251. DOI: 10.1002/adma.201102752

[3] Pirkanniemi, K.; Sillanpaa, M. Heterogeneous water phase catalysis as an environmental application: a review. *Chemosphere* **2002,** *48,* 1047-1060. DOI: 10.1016/S0045-6535(02)00168-6
